# CHSY3 promotes proliferation and migration in gastric cancer and is associated with immune infiltration

**DOI:** 10.1186/s12967-023-04333-x

**Published:** 2023-07-17

**Authors:** Xinkun Huang, Yonghui Liu, Chenyu Qian, Qicheng Shen, Menglong Wu, Bin Zhu, Ying Feng

**Affiliations:** 1 Department of Gastrointestinal Surgery, Affiliated Hospital of Nantong University, Medical School of Nantong University, Nantong, 226001 China; 2 Graduate School, Dalian Medical University, Dalian, 116000 Liaoning China; 3 Department of General Surgery, Yancheng First Hospital, Affiliated Hospital of Nanjing University Medical School, Yancheng, China; 4 Department of Central Laboratory, Yancheng First Hospital, Affiliated Hospital of Nanjing University Medical School, Yancheng, China; 5 Department of Laboratory Medicine, Nanjing Zhongda Hospital, School of Medicine, Southeast University, Nanjing, 210009 People’s Republic of China; 6 Medical school, Nantong University, 19 Qixiu Road, Nantong, 226001 Jiangsu China; 7 Research Center of Clinical Medicine, Affiliated Hospital of Nantong University, 20 Xisi Street, Nantong, 226001 Jiangsu China

**Keywords:** CHSY3, Gastric cancer, TIDE, IPS, Prognosis

## Abstract

**Background:**

The glycosyltransferase CHSY3 is a CHSY family member, yet its importance in the context of gastric cancer development remains incompletely understood. The present study was thus developed to explore the mechanistic importance of CHSY3 as a regulator of gastric cancer.

**Methods:**

Expression of CHSY3 was verified by TCGA, GEO and HPA databases. Kaplan–Meier curve, ROC, univariate cox, multivariate cox, and nomogram models were used to verify the prognostic impact and predictive value of CHSY3. KEGG and GO methods were used to identify signaling pathways associated with CHSY3. TIDE and IPS scores were used to assess the immunotherapeutic value of CHSY3. WGCNA, Cytoscape constructs PPI networks and random forest models to identify key Hub genes. Finally, qRT-PCR and immunohistochemical staining were performed to verify CHSY3 expression in clinical specimens. The ability of CHSY3 to regulate tumor was further assessed by CCK-8 assay and cloning assay, EDU assay, migration assay, invasion assay, and xenograft tumor model analysis.

**Results:**

The expression of CHSY3 was discovered to be abnormally upregulated in GC tissues through TCGA, GEO, and HPA databases, and the expression of CHSY3 was associated with poor prognosis in GC patients. Correlation analysis and Cox regression analysis revealed higher CHSY3 expression in higher T staging, an independent prognostic factor for GC. Moreover, elevated expression of CHSY3 was found to reduce the benefit of immunotherapy as assessed by the TIDE score and IPS score. Then, utilizing WGCNA, the PPI network constructed by Cytoscape, and random forest model, the Hub genes of COL5A2, POSTN, COL1A1, and FN1 associated with immunity were screened. Finally, the expression of CHSY3 in GC tissues was verified by qRT-PCR and immunohistochemical staining. Moreover, the expression of CHSY3 was further demonstrated by in vivo and in vitro experiments to promote the proliferation, migration, and invasive ability of GC.

**Conclusions:**

The results of this study suggest that CHSY3 is an important regulator of gastric cancer progression, highlighting its promise as a therapeutic target for gastric cancer.

**Supplementary Information:**

The online version contains supplementary material available at 10.1186/s12967-023-04333-x.

## Introduction

Gastric cancer (GC) is among the most prevalent forms of cancer, with over 1,000,000 diagnoses and 769,000 deaths annually throughout the world, making this the fifth most prevalent and fourth deadliest form of malignancy [1]. Prior studies have revealed many factors to influence the risk of GC development, including family history [2], diet [3, 4], alcohol intake, smoking [5, 6], and Epstein–Barr virus (EBV) infection [7]. The standard treatment for GC at present is radical tumor resection with perioperative chemotherapy when appropriate, while the standard of care for metastatic or unresectable GC includes chemotherapeutic regimens consisting of platinum-based agents, fluoropyrimidines, docetaxel, paclitaxel, and irinotecan [8–11]. While patients with early-stage GC who undergo surgery exhibit 90% 5-year overall survival (OS) rates, many patients are only diagnosed when the disease is relatively advanced and the opportunity for curative surgical intervention is no longer present [12]. Recent advances in multi-omic profiling efforts have led to the identification of many candidate prognostic biomarkers associated with specific cancer types, highlighting the promise of defining reliable molecular biomarkers associated with GC patient prognosis.

Most GC tumors are adenocarcinomas that can be further subdivided as per Lauren’s classification system into intestinal, diffuse, and intermediate types [13]. The World Health Organization (WHO) utilizes an alternative system in which these tumors are classified into tubular, papillary, mucinous (colloid), and poorly cohesive carcinoma subtypes, although the clinical value of these classification approaches is limited [14]. The Cancer Genome Atlas Research Network (TCGA) more generally classifies GC tumors based on their molecular characteristics into EBV-positive, chromosomally unstable, genome stable, and microsatellite unstable tumor subtypes [15], while the Asian Cancer Research Group (ACRG) has proposed a classification scheme based on the results of mRNA expression profiling, targeted sequencing, and somatic copy numbers that groups tumors into those exhibiting microsatellite instability (MSI), microsatellite stability (MSS)and an epithelial–mesenchymal transition phenotype (MSS/EMT), MSS and TP53+ (MSS/TP53+), or MSS/TP53− [16]. While these latter two systems rely on advanced molecular classification strategies, the underlying molecular pathogenic characteristics of these tumor types remain poorly characterized, underscoring the need for prognostic biomarker identification in order to guide individualized patient management and treatment.

Chondroitin sulfate (CS) synthases are enzymes responsible for CS polymerization that are commonly aberrantly expressed in specific cancer types. CHSY1 (CS synthase 1), for example, has been reported to be upregulated in GC wherein it functions as a promoter of proliferative, migratory, and invasive activity in addition to regulating apoptotic induction [17]. CHSY1 has also been reported to interact with the related glycosyltransferase CHSY3 (CS synthase 3) in the context of chondroitin polymerization [18]. CHSY3, which is encoded on chromosome 5q23.2, exhibits glucuronosyltransferase and *N*-acetylgalactosaminyl transferase activities [19]. IN recent reports, the expression of CHSY3 has been shown to be elevated in colorectal cancer and associated with poor patient outcomes [20]. However, the functional significance of CHSY3 in GC has yet to be characterized.

The current investigation clarifies the function and expression of CHSY3 in GC, revealing this gene to be upregulated in GC tissues. Such CHSY3 upregulation was significantly correlated with specific patient clinicopathological characteristics and associated with poor patient prognostic outcomes. Furthermore, abnormally elevated expression of CHSY3 reduces the benefit of immunotherapy, while functional experiments showed that CHSY3 can regulate the proliferation, migration, and invasive ability of GC cells. Together, these data highlight a potential role for CHSY3 as a promoter of GC development and underscore its potential relevance as a prognostic biomarker and pharmacological target in the therapy of GC.

## Materials and methods

### Data acquisition and processing

Gene expression data, associated mutations, and clinical data for GC patients were downloaded from The Cancer Genome Atlas (TCGA, https://portal.gdc.cancer.gov/) database. GSE66229, GSE65801, GSE63089, GSE54129, GSE51575, GSE26901, and GSE84433 datasets were downloaded from the Gene Expression Omnibus (GEO, https://www.ncbi.nlm.nih.gov/geo/) database. The expression of CHSY3 protein in GC was detected by querying the Human Protein Atlas (HPA, http://www.proteinatlas.org/) database for detection. Data underwent standardized preprocessing and log transformation using appropriate R packages, with differentially expressed genes being identified using the following criteria: |log2 fold change (FC)| ≥ 1 and False Discovery Rate (FDR) < 0.05.

### Time-dependent ROC and logistic regression analyses

After collecting patient outcome and CHSY3 gene expression data, receiver operating characteristic (ROC) curve analysis was carried out to determine OS at the 1-, 3-, and 5-year time points with the time ROC function. The resulting area under the curve (AUC) results were used to analyze the sensitivity and specificity of CHSY3 as a prognostic biomarker in GC. The association between CHSY3 expression levels and GC patient age, gender, pathological stage, T, N, and M stages was further assessed through logistic regression analyses. P < 0.05 was the significance threshold.

### Cox risk regression analyses

Univariate and multivariate Cox regression analyses were used to identify independent predictors of patient OS. Variables studied included pathological stage, T stage, N stage, and M stage, as well as CHSY3 expression.

### KEGG, GO and GSEA

The clusterProfiler package was utilized to perform KEGG and GO analysis according to the methods described in previous studies [21]. The Pi package was utilized to perform GSEA analysis according to the method described in previous studies [22].

### Immune infiltration analyses

The R ESTIMATE package was used to compute Stromal, Immune, and ESTIMATE scores for each patient tumor samples based on the observed gene expression profiles [23]. Furthermore, the correlation between CHSY3 and the percentage of different immune cells in GC was analyzed by the Cibersort [24].

### TIDE, IPS scores and MSI status analyses

The TIDE scores and MSI status of GC samples were calculated utilizing the Tumor Immune Dysfunction and Exclusion (TIDE, http://tide.dfci.harvard.edu/login/) database [25, 26]. Moreover, immunophenoscore (IPS) of GC patients were obtained in The Cancer Immunome Atlas (TCIA, https://tcia.at/home) database [27].

### Protein–protein interaction network construction

The relevant module genes selected by WGCNA [24] were used to obtain interaction information by STRING (https://string-db.org/) database, and the PPI network was constructed by Cytoscape software and searched for Hub genes.

### Cell culture

The AGS, MKN45, SGC7901, and HGC27 GC cell lines and the control GES-1 gastric mucosal cell line were obtained from GeneChem (Shanghai, China). The MFC cell were obtained from Pricella (Wuhan, China). All cells were grown in RPMI-1640 supplemented with FBS (10%) at 37 °C in a humidified 5% CO_2_ incubator.

### Clinical samples

68 paired tumor tissues and paracancerous samples were harvested from patients with a pathological diagnosis of GC who had not undergone any anti-tumor treatments prior to surgical resection at Nantong University Hospital from January 2010–December 2010. Patient follow-up ended in August 2015. Another cohort of 10 fresh GC tissues and adjacent normal tissues were collected from the same source for qRT-PCR assays between 2020 and 2021. The present investigation was approved by the ethical committee of the Affiliated Hospital of Nantong University, and all patients provided written informed permission.

### Cellular transfection

Lipofectamine 3000 (Invitrogen, USA) was used to transfect cells plated in 6-well plates with an shRNA specific for CHSY3 or a control construct purchased from GeneChem (Shanghai, China). Cells were utilized for downstream assays at 48 h post-transfection. Analyses were conducted in triplicate. CHSY3 overexpression plasmid was customized from GenePharma (Shanghai, China).

### qRT-PCR

TRIzol (Invitrogen, USA) was used to extract cellular RNA, after which qRT-PCR was conducted as described previously [28]. The primers used were listed as follows: CHSY3: F—AGTGGATGAGCGTGGCATTAGG; R—AGCAGCAGAGCGACCGTAGTAG; COL5A2: F—GGATCACAGGGACCA AGAGGAGAG; R—GCACCAGGTTGACCAGGAACAC; POSTN: F—GCGAGATCATCAAGCCAGCAGAG; R—TCCAGTCTCCAGGTTGTGTCAGG; COL1A1: F—GCCTCTGCTCTCCGACCTCTC; R—CTGCTTTGTGCTTTGGGAAGTTGTC; FN1: F—AGA GGCATAAGGTTCGGGAAGAGG; R—CGAGTCATCCGTAGGTTGGTTCAAG. GAPDH: F—TGCACCACAACTGCTTAGC; R—GGCATGGACTGTGGTCATGAG. Analyses were conducted in triplicate.

### Western blot assay and immunohistochemistry (IHC)

Western blotting and IHC were conducted in triplicate as per previously published protocols [29]. Anti-CHSY3 for Western blot was obtained from Abcepta (Beijing, China). Anti-CHSY3, anti-Granzyme B, anti-Perforin utilized for IHC was from Bioss (Beijing, China). Anti-PD-L1, anti-CD8, anti-CD4 and anti-Ki67 were purchased from Servicebio (Wuhan, China). Anti-Alpha Tubulin obtained from Protentech (Wuhan, China).

### Migration and invasion assay

Migration and invasion experiments were performed three times as described previously [30].

### EDU, CCK-8 and colony assay

DNA synthesis rate assessment was conducted utilizing Click-iT EDU Imaging Kits (Beyotime, Beijing, China), and experiments were finished with the method provided by the manufacturer. CCK-8 assay: 10 µl CCK-8 solution (Dojindo, Kumamoto, Japan) was added to 96-well plates at the time specified in the manufacturer’s instructions, which were incubated for 2 × 10^3^ cells, and then the absorbance was measured at 450 nm after continued incubation for 2 h at 37 °C. For the cloning experiments, crystal violet was utilized to stain the cell colonies, which were cultivated in 6-well plates for 14 days. All experiments were repeated three times.

### Experiments with animals

Male BALB/C nude mice aged 6 weeks purchased from the Animal Laboratory Center of Nantong University (Nantong, China). All animal experiments were approved by the Institutional Animal Care and Use Committee of Nantong University following the current guidelines for animal care and welfare. For the tumorigenicity studies, five million treated HGC-27 or AGS cells were subcutaneously injected 0.5 cm under the axilla of 6-week-old mice (n = 5 mice per group). Tumor volume was measured every 3 days and calculated as V = 0.5 × length × width^2^. The nude mice were sacrificed after 24 days, and the tumor tissues were extracted and collected for subsequent studies.

GeneChem (Shanghai, China) provided the purchase of Lv-shCHSY3, a modified slow virus, for this experimental study. To establish a subcutaneous tumor model of MFC, C57BL/6 mice were inoculated with different strains of MFC cells (1 × 10^6^) infected with the aforementioned slow virus. The administration of treatments began on the 9th day following tumor inoculation. At this point, the average tumor volume reached approximately 80 mm^3^ to 100 mm^3^. It is worth noting that the mice carrying the tumors were subjected to injections of PBS or anti-PD-L1 mAb (200 micrograms/mouse), with a dosage regimen of once every 3 days [31]. The anti-PD-L1 mAb used in this study was obtained from Bio X Cell. Tumor measurements were conducted every 3 days to monitor the growth and development of the tumors. The experimental endpoint was set at the 24th day after tumor cell injection or upon natural death of the mice. At the conclusion of the experiment, tumor volume and weight were measured, and tumor tissues were collected for further analysis and experimentation.

### Statistical analysis

The data are presented as means ± standard deviation (SD), and all experiments were repeated in triplicate. All statistical analyses were performed by GraphPad Prism 7.0 or SPSS (version 22.0) or R software (version 4.1.2). *P* < 0.05 was the threshold of significance. Statistical significance is described as follows ns, not significant; *P < 0.05; **P < 0.01; ***P < 0.001.

## Results

### Expression and prognosis analysis of CHSY3 in the database

**Fig. 1:**
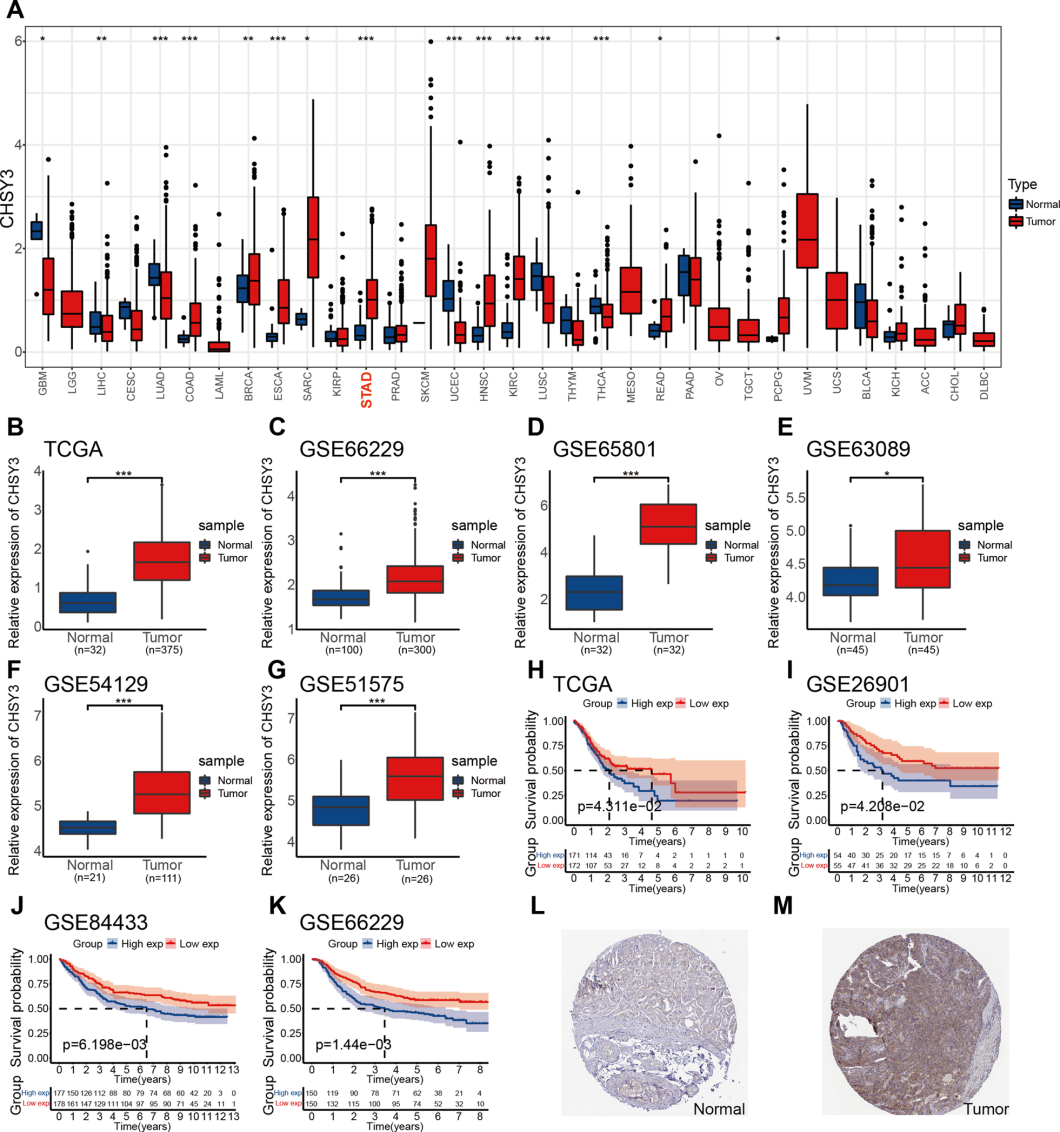
Expression and prognosis analysis of CHSY3 in the database. Expression of CHSY3 in pan-cancer from TCGA database (**A**). Relative expression levels of CHSY3 in 375 gastric cancer tissues and 32 normal tissues from TCGA database (**B**). Expression of CHSY3 in GSE66229 (N = 100, T = 300), GSE65801 (N = 32, T = 32), GSE63089 (N = 45, T = 45), GSE54129 (N = 21, T = 111), GSE51575 (N = 26, T = 26) datasets (**C**–**G**). Kaplan–Meier survival curve analysis of CHSY3 in GC from TCGA, GSE26901, GSE88433 and GSE66229 datasets (**H**–**K**). Immunohistochemical analysis of CHSY3 in gastric cancer by HPA database (**L**, **M**). *P < 0.05; **P < 0.01; ***P < 0.001

CHSY3 gene expression in stomach adenocarcinoma (GC) and healthy tissues from the TCGA database were analyzed, demonstrating increased expression of CHSY3 in tumors compared to normal tissues (Fig. 1A, B). The similar increase of CHSY3 was observed in GC tumor tissues in comparison to paired paracancerous tissues (Additional file 1: Fig. S1A). In addition, the expression results of CHSY3 in the datasets GSE66229, GSE65801, GSE63089, GSE54129, and GSE51575 were the same as those in the TCGA dataset (Fig. 1C–G). Kaplan–Meier curves indicated that patients expressing higher levels of CHSY3 exhibited a shorter OS relative to patients expressing lower levels of this glycosyltransferase (Fig. 1H–K), and time-dependent ROC curves assessing 1-, 3-, and 5-year OS as a function of CHSY3 expression levels yielded respective AUC values of 0.55, 0.63 and 0.70 (Additional file 1: Fig. S1B). IHC staining in the HPA database further confirmed the upregulation of CHSY3 at the protein level in GC (Fig. 1L, M). Together, these data indicate that CHSY3 is upregulated in GC and correlated with poor prognostic outcomes.

### CHSY3 expression correlates with patient clinicopathologic parameters

**Fig. 2:**
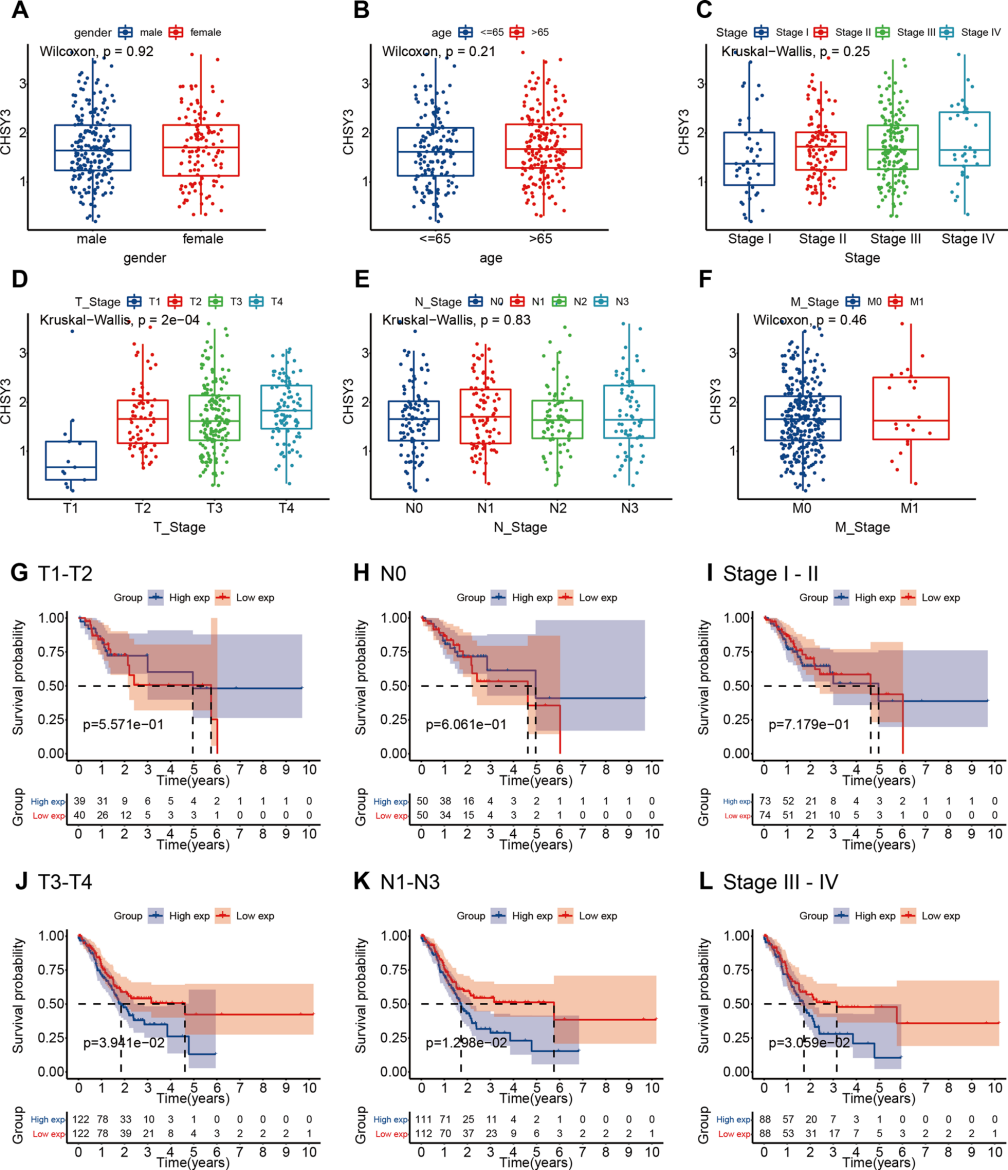
Association of CHSY3 expression with clinicopathologic parameters. Gender (**A**). Age (**B**). Stage (**C**). T (**D**). N (**E**). M (**F**). Analysis of KM survival curves in T1–2 patients (**G**). Analysis of KM survival curves in N0 patients (**H**). Analysis of KM survival curves in Stage I–II patients. **I** Analysis of KM survival curves in T3–4 patients (**J**). Analysis of KM survival curves in N1-3 patients. **K** Analysis of KM survival curves in Stage III–IV patients (**L**). *P < 0.05; **P < 0.01; ***P < 0.001

Associations between CHSY3 expression levels and patient clinicopathological characteristics in the TCGA database were analyzed, revealing higher levels of CHSY3 expression to be evident in GC patients with higher T stage. In contrast, CHSY3 expression was unrelated to GC patient gender, age, pathologic stage, N stage, or M stage (Fig. 2A–F). Subsequently, Kaplan–Meier curve analysis showed that CHSY3 expression was not associated with OS in GC patients with T1–2 stage, N0 stage, or Stage I–II. However, in T3–4 stage, N1–3 stage, or Stage III–IV, higher CHSY3 expression was associated with poor prognosis (Fig. 2G–L).

### CHSY3 is an independent predictor of patient outcome in GC and the construction of nomogram model

**Fig. 3:**
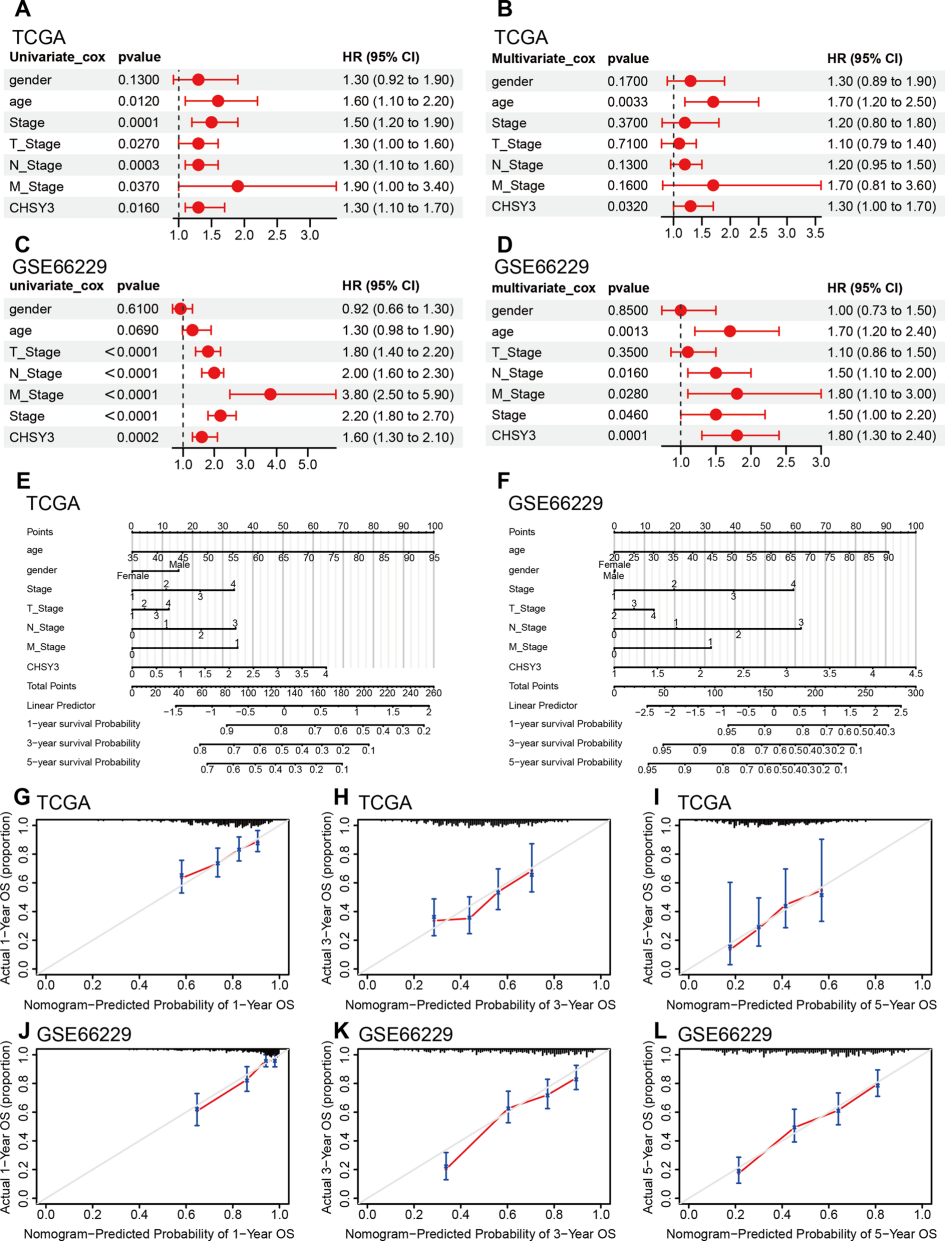
CHSY3 is an independent predictor of patient outcome in GC and the construction of nomogram model. Univariate and multivariate cox regression analysis in the TCGA (**A**, **B**) and GSE66229 datasets (**C**, **D**). Construction of a nomogram model of CHSY3 in TCGA (**E**) and GSE66229 (**F**) datasets. Calibration curves for the nomogram model for 1, 3, and 5 years (**G**–**L**)

The prognostic value of CHSY3 expression, pathological stage, and T/N/M stage in GC patients was assessed. Univariate Cox regression analysis and multivariate Cox regression analysis showed that CHSY3 was an independent prognostic factor for OS in GC (Fig. 3A, B). Moreover, in the GSE66229 dataset, for which complete clinical information is available, CHSY3 showed the same results (Fig. 3C, D).These findings indicated that CHSY3 expression offers value as an independent predictor of GC patient prognosis. Subsequently, we discovered that CHSY3 was a good predictor of patient prognosis at 1, 3, and 5 years by constructing a nomogram model (Fig. 3E, F). And the calibration curve also verified this (Fig. 3G–L).

### Functional analysis and mutational characterization of CHSY3

**Fig. 4:**
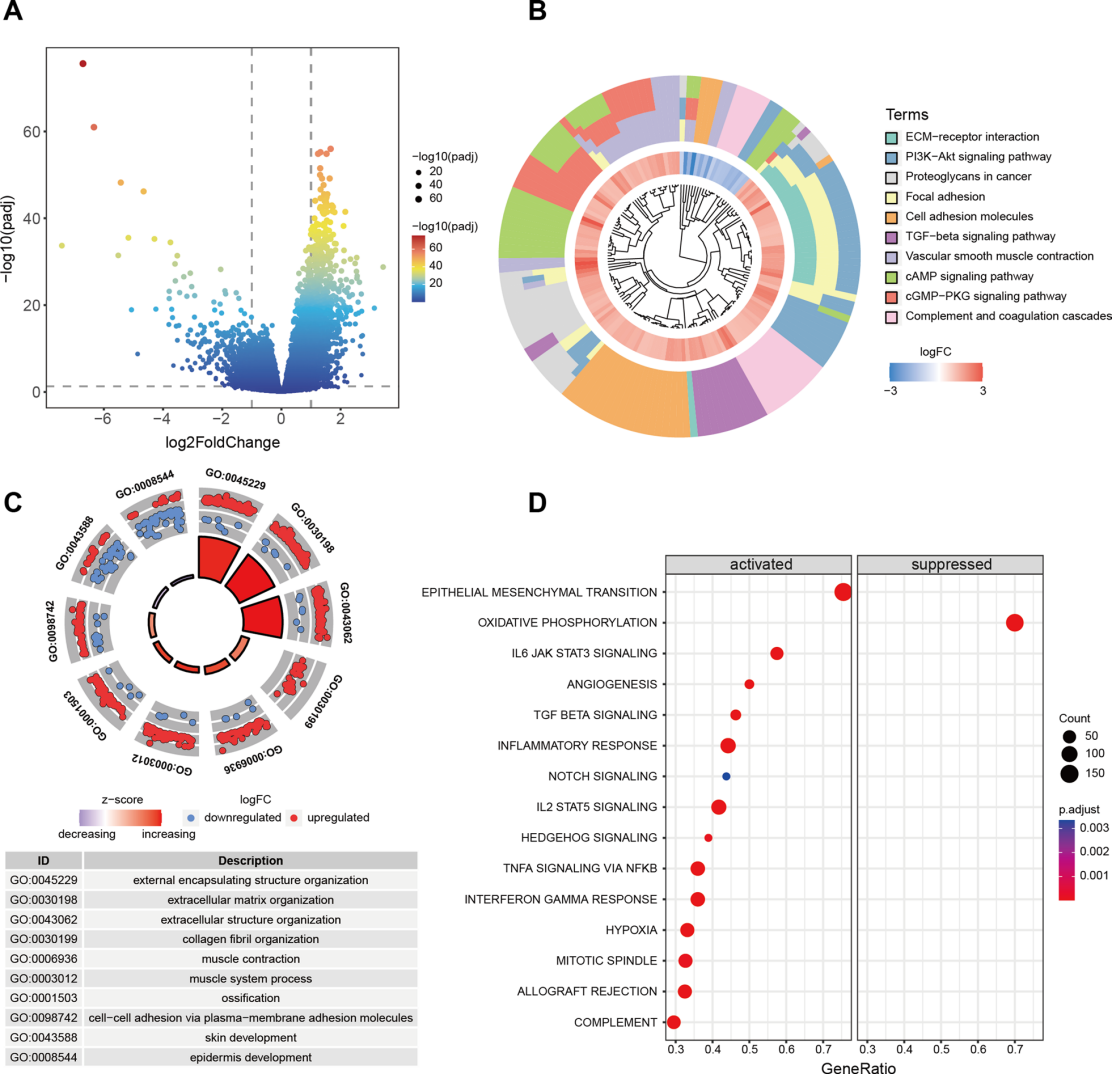
Functional analysis of CHSY3. Volcano plots represent differential gene expression between different CHSY3 expression subgroups (**A**). KEGG analysis of differential genes of CHSY3 (**B**). GO analysis (**C**). GSEA analysis (**D**)

We performed differential analysis of samples with high CHSY3 expression versus those with low CHSY3 expression and plotted volcanoes (Fig. 4A). The abnormally elevated CHSY3 expression was revealed by KEGG analysis to be enriched in signaling pathways such as ECM–receptor interaction, PI3K–Akt signaling pathway, TGF-beta signaling pathway, Complement and coagulation cascades, Cell adhesion molecules (Fig. 4B). GO analysis showed that CHSY3 is closely related to the functions of external encapsulating structure organization, extracellular matrix organization, extracellular structure organization, collagen fibril organization, and other functions are closely related (Fig. 4C). Furthermore, GSEA analysis revealed that CHSY3 expression was closely associated with the activation of Epithelial Mesenchymal Transition, angiogenesis, TGF-beta signaling, TNFa signaling via nfkb, Complement, and with the inhibition of Oxidative Phosphorylation (Fig. 4D and Additional file 2: Fig. S2). Additional file 3: Fig. S3A showed the mutational characteristics of different CHSY3 expression subgroups, and Additional file 3: Fig. S3B showed the co-occurrence and mutually exclusive relationships of the top 20 mutated genes. However, the expression of CHSY3 was not significantly associated with TMB in the TCGA database (Additional file 3: Fig. S3C).

### Immune infiltration analysis and analysis of the benefits of immunotherapy

**Fig. 5:**
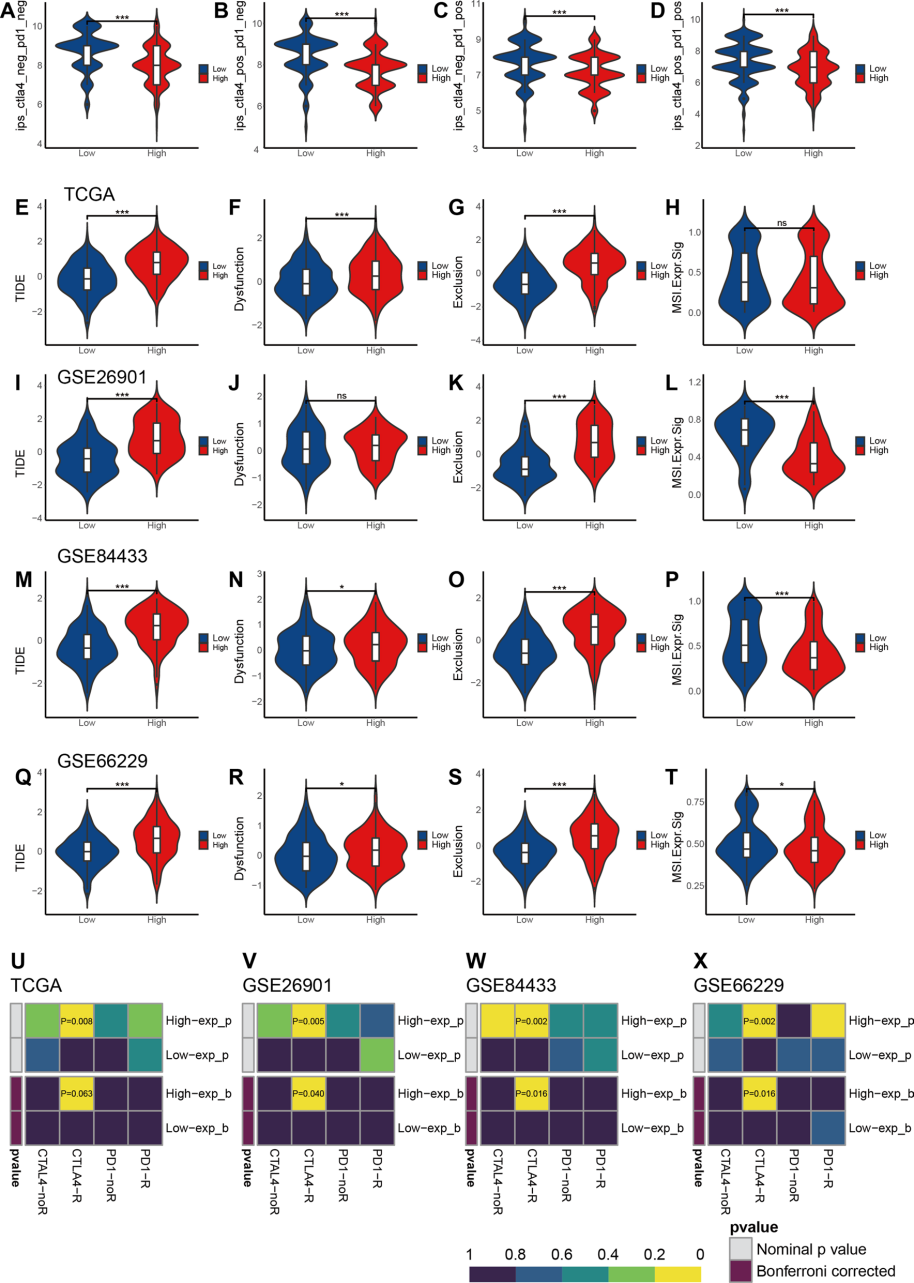
Analysis of CHSY3 expression on the benefits of immunotherapy. Correlation of IPS with CHSY3 expression (**A**–**D**). Correlation of CHSY3 expression with TIDE, dysfunction, exclusion, MSI in TCGA (**E**–**H**), GSE26901 (**I**–**L**), GSE84433 (**M**–**P**), GSE66229 (**Q**–**T**) datasets. Submap analysis of CHSY3 expression in relation to the sensitivity of anti-PD1 treatment and anti-CTLA4 treatment (**U**–**X**). *P < 0.05; **P < 0.01; ***P < 0.001

To investigate the mechanism by which elevated CHSY3 expression leads to poor prognosis in GC patients, we performed a Cibersort analysis and found that the percentage of Tregs cell infiltration was significantly higher in the low-CHSY3 group than in the high-CHSY3 group, while the percentage of M2 macrophage infiltration was significantly higher in the high-CHSY3 group than in the Low-CHSY3 group (Additional file 3: Fig. S3D). Subsequently, correlation analysis revealed that CHSY3 expression in GC was revealed to be significantly positively correlated with stromal, immune, and ESTIMATE scores, and negatively correlated with tumor purity (Additional file 3: Fig. S3E–H). The higher IPS, the more immunogenic the sample. Therefore, we analyzed the IPS scores of different CHSY3 expression groups by TCIA database, and the results showed that the IPS scores of low-CHSY3 group were higher than those of high-CHSY3 group, indicating that patients with low-CHSY3 expression had better efficacy for immunotherapy (Fig. 5A–D).

Then, we utilized TIDE to assess the potential immunotherapeutic efficacy of immunotherapy in different CHSY3-expressing subgroups. Higher TIDE prediction scores represent a higher likelihood of immune evasion, indicating that patients are less likely to benefit from ICI therapy [25]. In the TCGA dataset, high expression of CHSY3 had higher TIDE scores, Dysfunction scores, and Exclusion scores, however, MSI was not statistically different in the different CHSY3 subgroups (Fig. 5E–H). Furthermore, in the GSE26901, GSE84433, and GSE66229 datasets, which have sample numbers greater than 100, again revealed that the CHSY3 high expression group had higher TIDE scores. Interestingly, the MSI scores were higher in the low CHSY3 expression subgroup in these datasets (Fig. 5I–T). In addition, the results of the column association table generated from TIDE and submap are shown in Fig. 5U–X. Taken together, these results suggest that CHSY3 expression lead to reduce benefit to immunotherapy in GC patients.

### WGCNA analysis identifies genes associated with immunotherapy

**Fig. 6:**
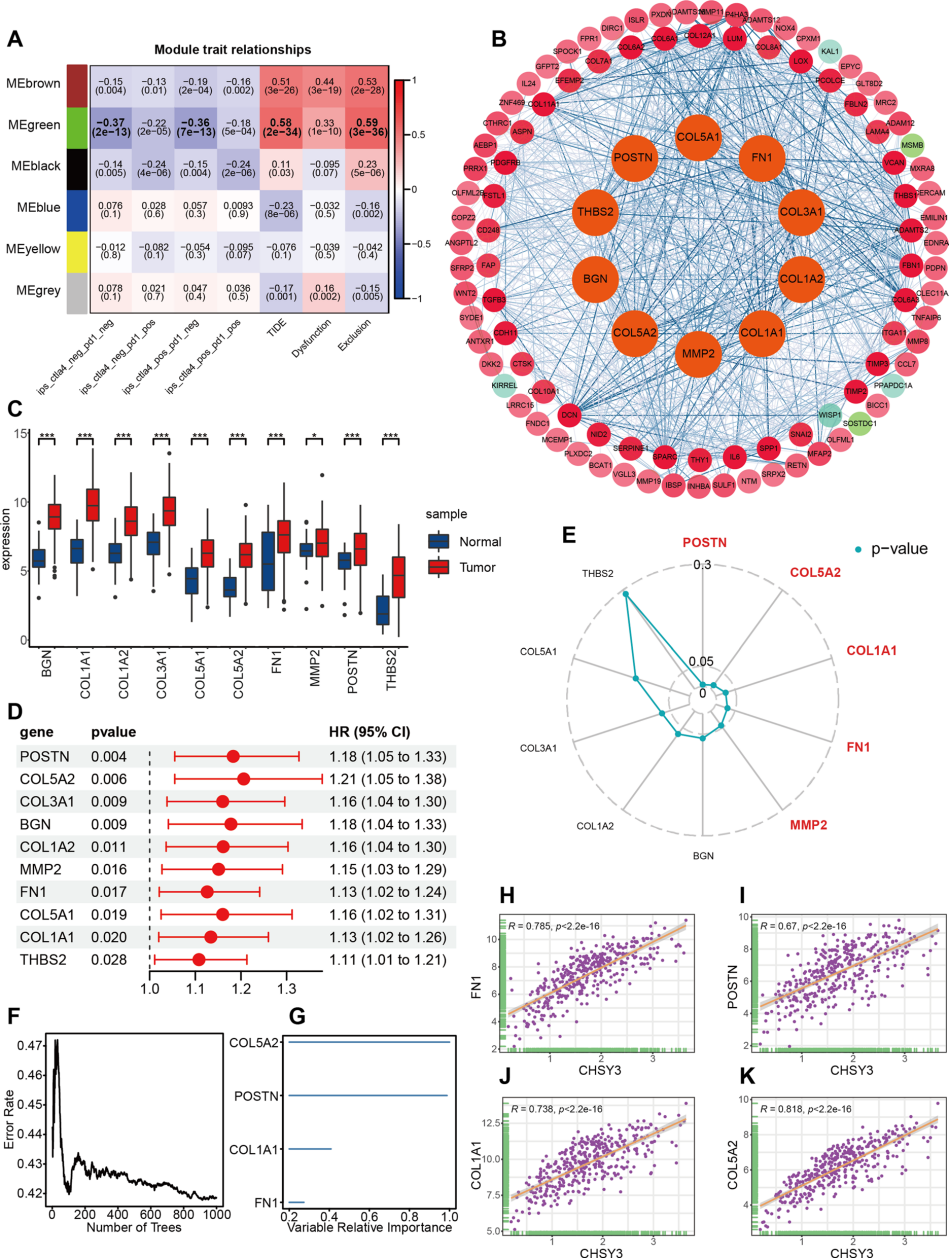
WGCNA analysis and construction of PPI network to identify Hub genes. Correlation of module signature genes with IPS and TIDE (**A**). Construction of PPI network to identify Hub genes (**B**). Expression of 10 Hub genes in GC tissues and paracancerous tissues in TCGA database (**C**). Univariate cox analysis of 10 Hub genes (**D**). Radar plot showing p-values of Kaplan–Meier survival curves for 10 Hub genes (**E**). Random Forests Identify Key Prognostic Genes (**F**, **G**). Scatter plot showing correlation of CHSY3 with FN1 (**H**), POSTN (**I**), COL1A1 (**J**), COL5A2 (**K**). *P < 0.05; **P < 0.01; ***P < 0.001

To explore the genes affecting immunotherapy, we performed WGCNA analysis in differential genes between different CHSY3 expression groups. The green module gene was negatively correlated with IPS scores and positively correlated with TIDE scores when power estimates were equal to 4 (Fig. 6A and Additional file 4: Fig. S4A–C). KEGG analysis showed that these genes were enriched in ECM–receptor interaction, TGF-beta signaling pathway, Toll-like receptor signaling pathway, PI3K–Akt signaling pathway, Wnt signaling pathway, and other signaling pathways (Additional file 4: Fig. S4D). And GO analysis showed that these genes were associated with extracellular matrix organization, ossification, collagen—containing extracellular matrix, fibrillar collagen trimer, extracellular matrix structural constituent, extracellular matrix binding, and other functions (Additional file 4: Fig. S4E–G).

### Construction of PPI network to identify hub genes

Subsequently, we constructed a PPI network to identify 10 Hub genes, namely BGN, COL1A1, COL1A2, COL3A1, COL5A1, COL5A2, FN1, MMP2, POSTN, and THBS2 (Fig. 6B). In the TCGA database, all 10 hub genes were highly expressed in gastric cancer compared to paired non-cancerous tissues, and univariate cox analysis showed that the HR of all these genes was greater than 1 (Fig. 6C, D). Besides, high expression of five genes, POSTN, COL5A2, COL1A1, FN1, and MMP2, was revealed by Kaplan–Meier survival curve analysis to suggest a poor prognosis for gastric cancer patients (Fig. 6E and Additional file 5: Fig. S5). Then, the effect of POSTN, COL5A2, COL1A1, FN1, and MMP2 on GC prognosis was identified by random forest model analysis. The genes with variable relative importance greater than 0 were COL5A2, POSTN, COL1A1, and FN1, thus we concluded that these 4 Hub genes had the greatest impact on the prognosis of GC patients (Fig. 6F, G). Scatter plots revealed a significant positive correlation between the expression of COL5A2, POSTN, COL1A1, and FN1 and the expression of CHSY3 (Fig. 6H–K).

### GC tissues exhibit CHSY3 upregulation correlated with poor prognostic outcomes

**Fig. 7:**
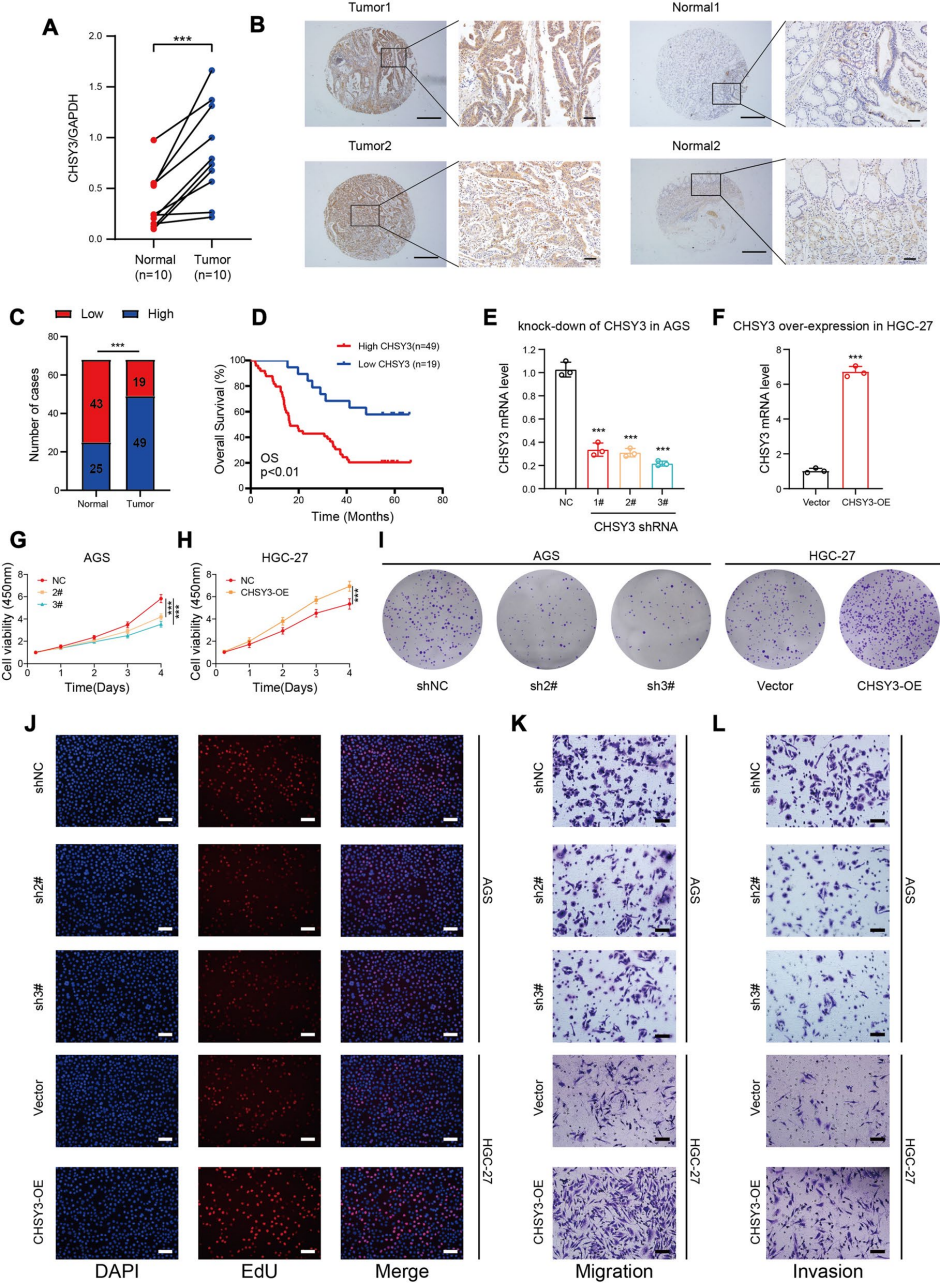
CHSY3 regulates GC cell proliferation, migration and invasion. qRT-PCR analysis of the relative expression of CHSY3 in 10 pairs of GC samples and normal gastric tissues (**A**). Immunohistochemical analysis of CHSY3 in 68 on GC tissue and normal tissue (**B**, **C**). KM curve analysis of 68 GC samples (**D**). Knockdown efficiency (**E**) and overexpression efficiency (**F**) of CHSY3 in GC cells. CCK-8 assay analysis (**G**, **H**). Cloning experiment analysis (**I**). EDU assay analysis (**J**). Migration assay analysis (**K**). Invasion assay analysis (**L**). *P < 0.05; **P < 0.01; ***P < 0.001

To confirm the above findings, qRT-PCR analyses of additional GC patient tumor samples were conducted, revealing CHSY3 to be upregulated in 10 GC tumor samples relative to levels in paired paracancerous samples (Fig. 7A). This finding was confirmed by a tissue microarray examination of 68 pairs of paracancerous and tumor tissues from GC patients, with 49/68 patients exhibiting increased intratumoral CHSY3 expression whereas 19 exhibited lower CHSY3 protein levels in tumor samples (Fig. 7B, C). In addition, correlations between CHSY3 expression and GC patient clinicopathological characteristics were examined, revealing higher levels of CHSY3 expression to be correlated with depth of invasion, advanced TNM stage, and lymph node metastasis (Table 1). Univariate and multivariate analyses confirmed that CHSY3 expression was an independent predictor of the OS of GC patients (Table 2). Additionally, these patients were divided into CHSY3-high and CHSY3-low groups, with Kaplan–Meier analyses revealing that CHSY3 expression is related to a poorer patient prognosis (Fig. 7D).

**Table 1:** Correlation between CHSY3 expression in GC tissues and clinicopathological features of GC patients

Clinicopathological Parameter	N	CHSY3 expression		p-value
		Low 19	High 49	
Gender				0.683
Male	42	11	31	
Female	26	8	18	
Age (years)				0.72
< 65	37	11	26	
≥ 65	31	8	23	
Degree of differentiation				0.27
Well	3	0	3	
Moderate/poor	65	19	46	
Tumor diameter (cm)				0.938
< 5	47	13	34	
≥ 5	21	6	15	
TNM stage				< 0.001
I + II	31	16	15	
III	37	3	34	
Depth of invasion				0.015
T1 + T2	24	11	13	
T3 + T4	44	8	36	
Lymph node metastasis				0.026
Negative	22	10	12	
Positive	46	9	37	

**Table 2:** Univariate and multivariable analysis of OS of patients with GC

OS	Univariate analysis	Multivariable analysis	
	P > |z|	P > |z|	HR (95% CI)
Gender	0.203		
Male (n = 42) vs. female (n = 26)			
Age (years)	0.580		
< 65 (n = 37) vs. ≥ 65 (n = 31)			
Differentiation	0.526		
Well (n = 3) vs. moderate/poor (n = 65)			
Tumor diameter (cm)	0.061		
< 5 (n = 47) vs. ≥ 5 (n = 21)			
Depth of invasion	0.001	0.033	2.225 (1.068–4.633)
T1 + T2 (n = 24) vs. T3 + T4 (n = 44)			
Lymph node metastasis	< 0.001	0.034	2.392 (1.068–5.360)
Negative (n = 22) vs. positive (n = 46)			
CHSY3 expression	0.003	0.045	2.287 (1.020–5.129)
Low (n = 19) vs. high (n = 49)			

### CHSY3 regulates GC cell proliferation, migration and invasion

Lastly, the functional role of CHSY3 was explored in GC cells. Initially, CHSY3 mRNA levels were analyzed in GC cell lines, revealing it to be maximally upregulated in AGS cells and to a lesser extent in HGC-27 cells (Additional file 6: Fig. S6A, B). Then, CHSY3 was knocked down in AGS cells and overexpressed in HGC-27 cells (Fig. 7E, F and Additional file 6: Fig. S6C, D). Meanwhile, qRT-PCR assays of COL5A2, POSTN, COL1A1, and FN1 showed that the expression of these four genes decreased when CHSY3 was knocked down, while their expression increased when CHSY3 was overexpressed (Additional file 6: Fig. S6E–L). Next, CCK-8 showed that the knockdown of the CHSY3 was sufficient to inhibit GC cell viability, while overexpression of the CHSY3 promoted GC cell viability (Fig. 7G, H). In addition, cloning assays and EDU proliferation assays demonstrated the same results, where knockdown of CHSY3 inhibited the proliferation of GC cells, while overexpression of CHSY3 promoted the proliferation of GC cells (Fig. 7I, J). Finally, Transwell assay additionally indicated that CHSY3 knockdown was sufficient to inhibit GC cell migration and invasion relative to that of control cells, while upregulation of CHSY3 promoted the migration and invasion of GC cells (Fig. 7K, L). In summary, knockdown of CHSY3 impairs the proliferation, migration, and invasion of gastric cancer cells.

### CHSY3 promotes tumor growth in nude mice

**Fig. 8:**
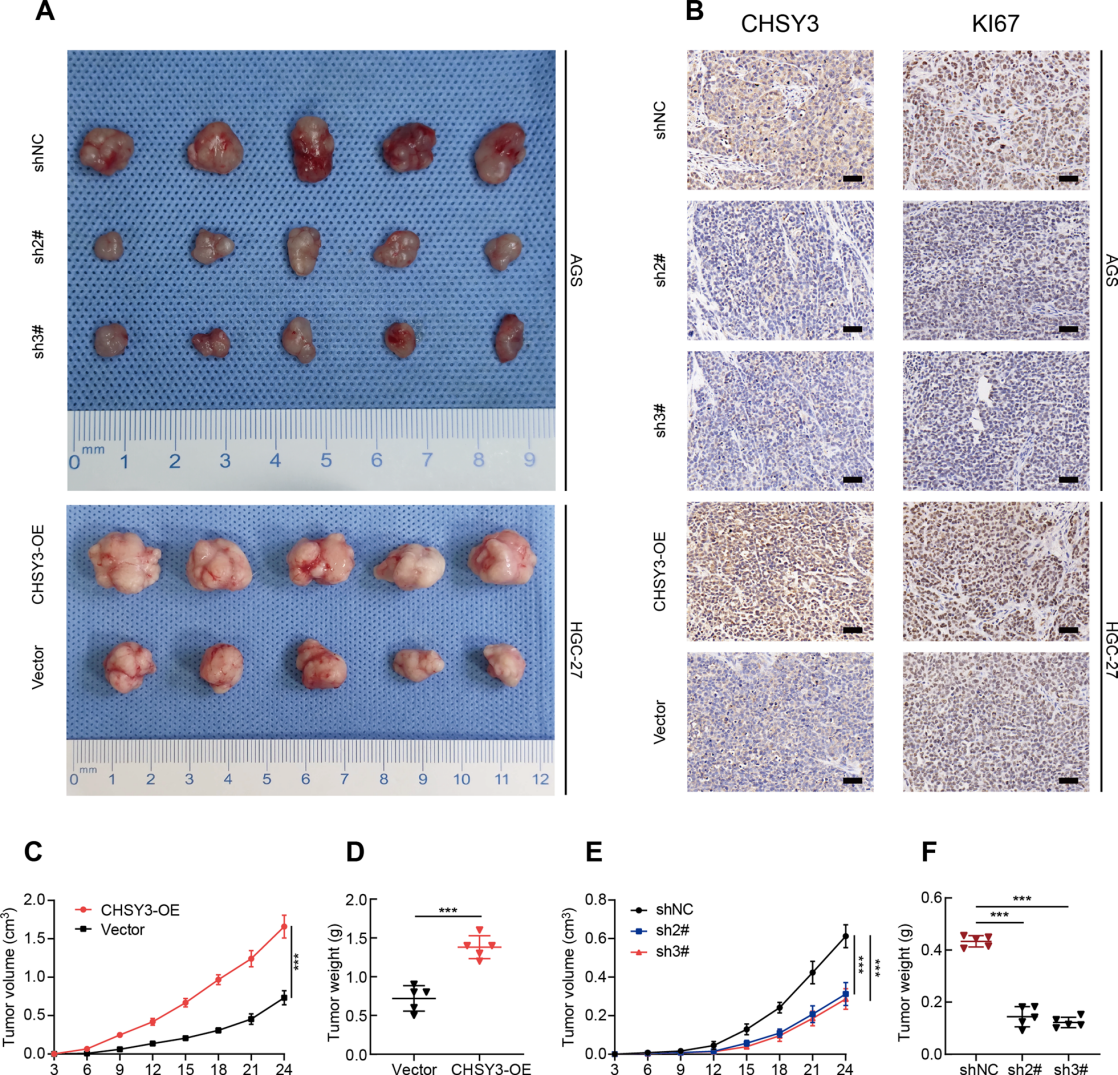
CHSY3 promotes tumor growth in nude mice. Tumor growth of mice implanted subcutaneously with GC cells that have undergone overexpression of CHSY3 (**A**). Immunohistochemistry revealed the expression of CHSY3 and Ki67 in subcutaneously transplanted tumors in mice (Scale: 40 μm) (**B**). Tumor volume and weight were measured to show tumor size (**C**, **D**). *P < 0.05; **P < 0.01; ***P < 0.001

In subcutaneous tumor experiments in nude mice, tumor growth was significantly faster in nude mice injected with CHSY3 overexpressing HGC-27 cells than in controls, while tumor growth was significantly slower in nude mice injected with CHSY3 knockdown AGS cells than in controls (Fig. 8A). Immunohistochemical staining showed that Ki67 expression was significantly upregulated in the CHSY3-OE group compared to the vector group, while Ki67 expression was significantly suppressed in the CHSY3 knockdown group compared to the negative control group (Fig. 8B). The volume and weight of subcutaneous tumors in the CHSY3-OE group were significantly larger than those in the carrier group, while the volume and weight of subcutaneous tumors in the CHSY3 knockdown group were significantly smaller than those in the negative control group (Fig. 8C–F).

### Expression of CHSY3 and chemotherapeutic drug sensitivity analysis

Chemotherapy is one of the most critical treatments for GC. The R package ‘pRRophetic’ is utilizing the expression matrix and drug handling information inside the Cancer Genome Project (CGP) program, a database with information on 138 anticancer drugs [32]. In TCGA, GSE26901, GSE84433, and GSE66229 datasets, we discovered that CHSY3 expression was negatively correlated with drug sensitivity of cisplatin and docetaxel, which provided us with favorable assistance in the subsequent combination therapy of GC patients (Additional file 7: Fig. S7A, B).

### In vivo antitumor effects of CHSY3 knockdown combined with αPD-L1

**Fig. 9:**
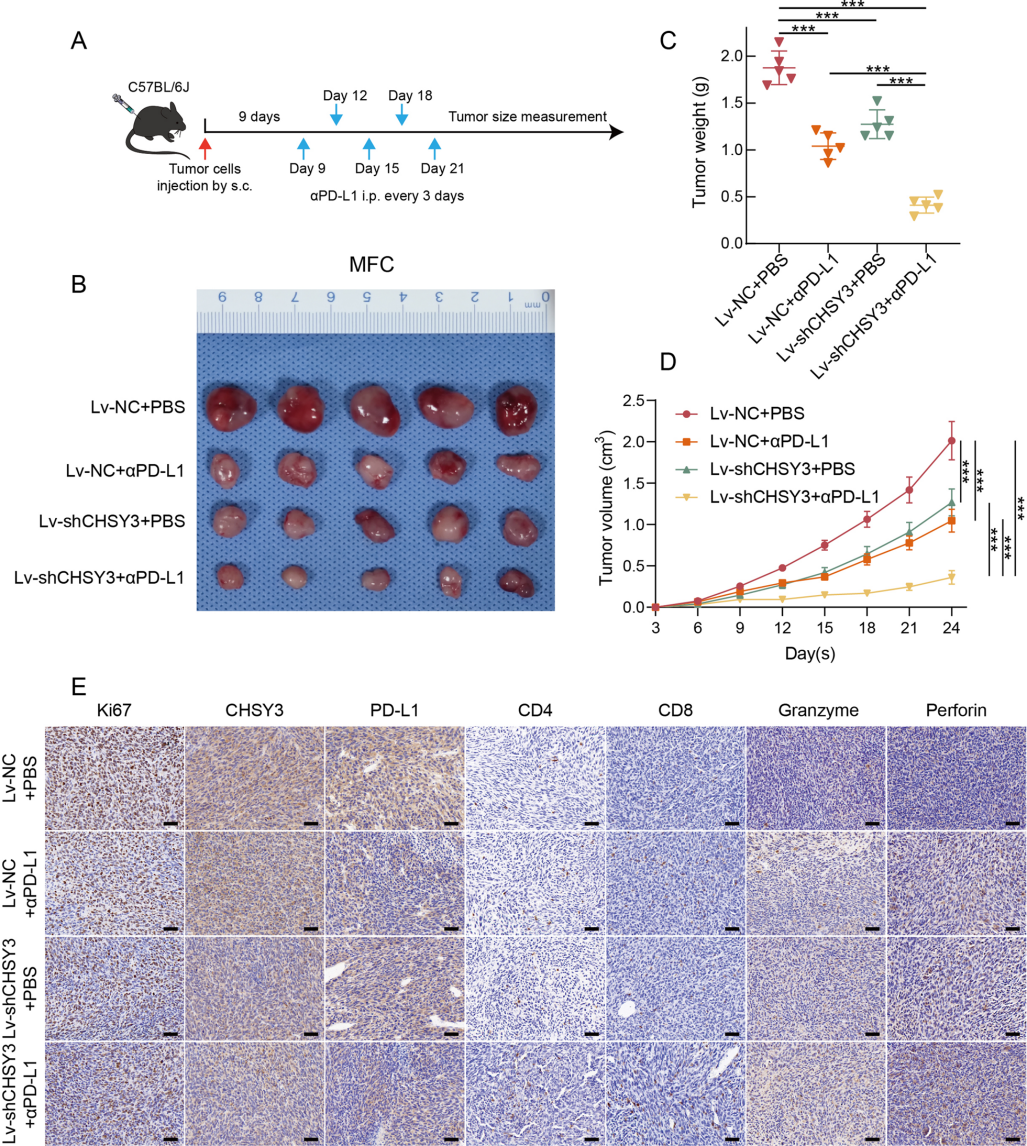
In vivo antitumor effects of CHSY3 Knockdown Combined with αPD-L1. Therapeutic regimen for tumor-bearing mice (**A**). Images of isolated tumours from MFC tumour-bearing mice (**B**). Tumor growth curve and tumor weight in MFC tumor-bearing mice (**C**, **D**). Immunohistochemistry of MFC tumors (scale: 40 μm) (**E**). *P < 0.05; **P < 0.01; ***P < 0.001

The antineoplastic efficacy of CHSY3 inhibition in combination with αPD-L1 was evaluated in an MFC tumour model where mice were subjected to subcutaneous injection of tumour cells. After 9 days, the mice were divided into different groups and subjected to different treatments, as shown in Fig. 9A. The CHSY3 knockdown group, as well as the αPD-L1 group, showed partial inhibition of tumour growth in the MFC tumour model. However, when subjected to the combined treatment regimen, a remarkable antineoplastic effect was observed as shown by the data in Fig. 9B. Furthermore, the tumour volume curve and tumour weight were consistent with the tumour suppressive effect of the combined treatment as shown in Fig. 9C, D respectively. To further explore the differences in the immune milieu following the synergistic intervention of CHSY3 inhibition and αPD-L1, immunohistochemistry focusing on CD8+ T cells within MFC tumours was performed. Notably, the immunohistochemical images shown in Fig. 9E clearly demonstrate the strong increase in CD8+ T cell infiltration resulting from the combined treatment modality. Additionally, the upregulation of Granzyme B and Perforin, as indicated, suggests an enhanced activation of CD8+ T cells. These findings indicate that the combined intervention effectively promotes CD8+ T cell activity.

## Discussion

GC is one of the most common and lethal types of cancer in the world. While surgical resection is often a curative option for early-stage GC patients, those with more advanced disease must undergo chemotherapy and exhibit poor 5-year survival rates [33, 34]. Efforts to enhance the efficacy of chemotherapy or to improve targeted disease treatment and prognostic management efforts are thus critical to ensuring GC patients experience better outcomes. Recent advances in genetic sequencing and associated technologies have highlighted new approaches to clarifying the molecular mechanisms governing GC development and progression, leading to the detection of novel targets for potential pharmacological intervention in this oncological context [35].

In this study, the role of CHSY3 in GC was thoroughly investigated using RNA expression data from the TCGA database and GEO Database. CHSY3 upregulation was observed in GC tumor tissues, and patients expressing higher levels of this gene exhibited poorer prognostic outcomes. Consistently, IHC data from the HPA database confirmed the upregulation of CHSY3 in GC tumors relative to normal tissues. Higher levels of CHSY3 expression were also evident in patients with more advanced GC. These findings are consistent with prior data demonstrating CHSY3 upregulation in rectal cancer to be associated with worse patient outcomes [20]. CHSY3 was found to be an independent predictor of poor GC patient prognosis in subsequent univariate and multivariate Cox regression analyses.

The immune surveillance model describes a well-accepted theory wherein immune cells can recognize and eliminate tumors and other neoplastic cells [36]. The ability of tumors to avoid immune-mediated destruction can thus occur through elimination, equilibrium, and escape mechanisms [37, 38], with tumor development often proceeding rapidly following immune evasion. Immunotherapeutic treatments for cancers rely on the use of antibodies or immune tumor vaccines to better engage host anti-tumor immune responses so as to promote tumor clearance [39]. The potential relationship between CHSY3 expression and immune status was examined using the CIBERSOR approach, revealing it to be correlated with Tregs, M2macrophages, and resting Mast cells. Previous studies have shown that macrophage M2 polarization plays an important role in the development of oral squamous cell carcinoma, colorectal cancer, and gastric cancer [40–42]. CHSY3 levels were also significantly related to the composition of the tumor microenvironment, as evidenced by their significant correlation with immune, stromal, and ESTIMATE scores. Recent data support a close link between GC patient TMB/MSI status and prognostic outcomes [43–45]. However, this study found no statistically significant association between CHSY3 levels and TMB status in GC patients. Interestingly, although there was no relationship between CHSY3 expression and MSI status in the TCGA dataset, the CHSY3 low expression group exhibited higher MSI scores in the GSE26901, GSE84433, and GSE66229 independent datasets. Subsequently, we also assessed the association of CHSY3 expression with immunotherapy by TIDE score and IPS score, and the results showed that CHSY3 expression was negatively correlated with the effect of immunotherapy. To better determine the mechanism by which CHSY3 affects immunotherapy, we screened the gene modules associated with TIDE score and IPS score from within the differential genes by WGCNA analysis. In addition, 10 Hub genes, BGN, COL1A1, COL1A2, COL3A1, COL5A1, COL5A2, FN1, MMP2, POSTN, and THBS2, were screened by the construction of PPI network. Finally, through KM curve survival analysis combined with random forest model, we concluded that POSTN, COL5A2, COL1A1, and FN1 may be the 4 most critical genes and these 4 genes have a strong positive correlation with CHSY3. Together, these data provide a promising foundation for the immunotherapeutic treatment of GC, suggesting differential CHSY3 expression to be of potential relevance in the context of GC patient immune response status.

Through additional analyses of 10 pairs of GC patient paracancerous and tumor tissues collected, the upregulation of CHSY3 in GC was further confirmed in an independent dataset. Similar results were obtained for tissue microarrays composed of tumors from 68 GC tissues and normal gastric tissue from 68 GC patients, and Kaplan–Meier analysis showed that higher levels of CHSY3 expression were similarly associated with poor prognosis in this group of GC tumor tissues. In addition, further experiments were performed to assess the importance of CHSY3 for GC cells by knocking down the expression of CHSY3 in AGS cells and overexpressing CHSY3 in HGC-27 cells. Furthermore, the regulation of POSTN, FN1, COL1A1, and COL5A2 by CHSY3 was confirmed by qRT-PCR. POSTN was reported to enhance the resistance of glioma stem cells to anti-angiogenic therapy by positively regulating VEGFA expression through activation of STAT3 [46]. FN1 was reported to promote invasive metastasis in papillary thyroid cancer due to its activation by the nf-κb signaling pathway [47]. In GC, COL1A1 can promote tumor progression as a promising prognostic target [48]. Additionally, COL5A2 may be a potential clinical biomarker for GC metastasis [49]. In subsequent CCK-8 assays, cloning assays, and EDU assays, it was observed that knockdown of CHSY3 was sufficient to inhibit GC cell proliferation, while aberrant expression of CHSY3 was able to promote GC cell proliferation. Our group also supplemented migration and invasion experiments to demonstrate that CHSY3 expression regulates GC cell migration and invasion ability. In nude mice with subcutaneous xenograft tumors, our group found similar results, where subcutaneous injection of GC cells overexpressing CHSY3 effectively promoted the growth of subcutaneous tumors in nude mice, while subcutaneous injection of GC cells knocking down CHSY3 effectively inhibited the growth of subcutaneous tumors in nude mice. Taken together, our database analysis and related experimental results are sufficient to demonstrate that CHSY3 expression promotes GC proliferation, migration, and invasive ability.

In the realm of contemporary medical advancements, despite the fact that immune checkpoint inhibitor (ICI) therapy has emerged as an optimal therapeutic approach for select cases in oncology, endowing previously unprecedented extensions to survival periods within certain patient cohorts, the efficacy and applicability of this approach are hindered by the lamentable emergence of resistance phenomena, be it intrinsic or acquired, pertaining to immune-based treatments [50, 51]. Based on an abundance of meticulously conducted clinical investigations, it has been irrefutably established that only a fraction of afflicted individuals derive the long-term beneficial responses sought after, with the disheartening experience of immunotherapy resistance encompassing the vast majority. In a valiant endeavor to surmount this perplexing conundrum, the exploration and validation of multifaceted regimens involving the employment of combination immunotherapies in the context of combating various forms of malignancies have gained immense recognition and garnered substantial interest [52]. It is within this intricate landscape that we unveil our groundbreaking revelation, whereby the synergistic alliance of targeting CHSY3 alongside αPD-L1 interventions evinces the conspicuous augmentation of immune cell infiltration within tumor microenvironments, thus instigating a commensurate elevation in the therapeutic efficacy of immune-based treatments.

By utilizing a bioinformatics approach, this study offers valuable insights into biological systems with the aim of predicting the relationship between CHSY3 and the regulation of antitumor immune responses. Additionally, laboratory pre-experiments have demonstrated that suppressing CHSY3 can enhance the therapeutic effects of anti-PD-L1 treatment. Subsequent in vivo experiments have effectively validated the validity of targeting CHSY3, thereby demonstrating its potential to improve the effectiveness of anti-PD-L1 therapeutic interventions. However, it is crucial to recognize the limitations associated with the relatively small sample size in the TCGA database and the use of independent samples in this study. These factors may introduce inherent bias into the outcome data. Consequently, it becomes evident that extensive large-scale prospective studies are necessary prerequisites for confirming and expanding upon these observations.

## Conclusion

Taking into consideration the aforementioned discourse, the current analysis substantiates CHSY3 as a highly promising prognostic biomarker and an effective therapeutic target in the context of gastric cancer (GC). The upregulation of this particular glycosyltransferase exhibits a significant correlation with unfavorable overall survival (OS) in GC patients, as well as higher pathological staging and T-stage outcomes. Further correlation analysis between the Tumor Immune Dysfunction and Exclusion (TIDE) score and the Immune Prognostic Score (IPS) suggests a compelling association with CHSY3 expression, thereby impairing the efficacy of immune-based therapies. Moreover, employing an integrative approach encompassing Weighted Gene Co-expression Network Analysis (WGCNA) and Cytoscape, we have successfully identified ten hub genes intricately linked to immune therapy and gastric cancer progression. Among these, COL5A2, POSTN, COL1A1, and FN1 emerge as potentially pivotal candidates warranting further investigation. Notably, in both in vitro and in vivo experiments, modulation of CHSY3 expression adequately regulates the proliferative, migratory, and invasive capabilities of gastric cancer cells. Furthermore, in vivo experiments have unequivocally demonstrated that the targeted inhibition of CHSY3, in combination with anti-PD-L1 therapy, significantly suppresses tumor growth. Collectively, these compelling findings underscore novel therapeutic avenues for the management of gastric cancer, although meticulous scrutiny is essential to unravel the intricate molecular mechanisms underpinning these observations.

## Supplementary Information

**Additional file 1: Figure S1.** Expression of CHSY3 and ROC curve. Relative expression levels of CHSY3 in 27 pairs of gastric cancer tissues and matched paracancerous normal tissues in the TCGA database (A). The 1-year, 3-year, and 5-year ROC curves of CHSY3 (B). *P < 0.05; **P < 0.01; ***P < 0.001.

**Additional file 2: Figure S2.** GSEA analysis of CHSY3.

**Additional file 3: Figure S3.** Mutation characteristics and immunological characteristics of CHSY3. Waterfall diagram showing CHSY3 mutation characteristics (A). Correlation between the top 20 genes with mutation frequency in different CHSY3 expression subgroups (B). Association between different CHSY3 expression subgroups and TMB with a threshold of 10 muts/Mb (C). Cibersort analysis of different immune cell proportions (D). Correlation of CHSY3 expression with immune score (E), stromal score (F), ESTIMATE score (G), and tumor purity (H). *P < 0.05; **P < 0.01; ***P < 0.001.

**Additional file 4: Figure S4.** WGCNA and functional analysis. GC sample clustering in the TCGA database (A). Topological network analysis to identify optimal soft thresholds (B). Module identification (C). Kyoto encyclopedia of genes and genomes analysis (D). Gene ontology analysis (E–G).

**Additional file 5: Figure S5.** Kaplan–Meier survival curve analysis of 10 Hub genes.

**Additional file 6: Figure S6.** Verification of CHSY3 expression. The expression of CHSY3 in gastric cancer cell lines was analyzed by qRT-PCR and Western blot (A, B). Western blot to verify CHSY3 knockdown efficiency and overexpression efficiency (C, D). Relative mRNA expression of POSTN, FN1, COL1A1 and COL5A2 in AGS cells after CHSY3 knockdown (E–H). Relative mRNA expression of POSTN, FN1, COL1A1 and COL5A2 after overexpression of CHSY3 in HGC-27 cells (I–L). *P < 0.05; **P < 0.01; ***P < 0.001.

**Additional file 7: Figure S7.** Analysis of CHSY3 expression and chemotherapeutic drug sensitivity. The R package ‘pRRophetic’ analyzed the relationship between CHSY3 expression and cisplatin sensitivity (A). Relationship between CHSY3 and docetaxel sensitivity (B). *P < 0.05; **P < 0.01; ***P < 0.001.

## Data Availability

The datasets utilized or analyzed in the current study are available from the corresponding authors at reasonable request.

## References

[1] Sung H, Ferlay J, Siegel RL, Laversanne M, Soerjomataram I, Jemal A, Bray F (2021). Global cancer statistics 2020: GLOBOCAN estimates of incidence and mortality worldwide for 36 cancers in 185 countries. CA Cancer J Clin.

[2] Yaghoobi M, Bijarchi R, Narod SA (2010). Family history and the risk of gastric cancer. Br J Cancer.

[3] Kim J, Cho YA, Choi WJ, Jeong SH (2014). Gene-diet interactions in gastric cancer risk: a systematic review. World J Gastroenterol.

[4] Keszei AP, Goldbohm RA, Schouten LJ, Jakszyn P, van den Brandt PA (2013). Dietary N-nitroso compounds, endogenous nitrosation, and the risk of esophageal and gastric cancer subtypes in the Netherlands cohort study. Am J Clin Nutr.

[5] Moy KA, Fan Y, Wang R, Gao YT, Yu MC, Yuan JM (2010). Alcohol and tobacco use in relation to gastric cancer: a prospective study of men in Shanghai, China. Cancer Epidemiol Biomark Prev.

[6] Duell EJ, Travier N, Lujan-Barroso L, Clavel-Chapelon F, Boutron-Ruault MC, Morois S, Palli D, Krogh V, Panico S, Tumino R (2011). Alcohol consumption and gastric cancer risk in the European prospective investigation into cancer and nutrition (EPIC) cohort. Am J Clin Nutr.

[7] Camargo MC, Murphy G, Koriyama C, Pfeiffer RM, Kim WH, Herrera-Goepfert R, Corvalan AH, Carrascal E, Abdirad A, Anwar M (2011). Determinants of Epstein–Barr virus-positive gastric cancer: an international pooled analysis. Br J Cancer.

[8] Japanese Gastric Cancer A (2017). Japanese gastric cancer treatment guidelines 2014 (ver. 4). Gastric Cancer.

[9] Sasako M, Sakuramoto S, Katai H, Kinoshita T, Furukawa H, Yamaguchi T, Nashimoto A, Fujii M, Nakajima T, Ohashi Y (2011). Five-year outcomes of a randomized phase III trial comparing adjuvant chemotherapy with S-1 versus surgery alone in stage II or III gastric cancer. J Clin Oncol.

[10] Ajani JA, Lee J, Sano T, Janjigian YY, Fan D, Song S (2017). Gastric adenocarcinoma. Nat Rev Dis Prim.

[11] Koizumi W, Narahara H, Hara T, Takagane A, Akiya T, Takagi M, Miyashita K, Nishizaki T, Kobayashi O, Takiyama W (2008). S-1 plus cisplatin versus S-1 alone for first-line treatment of advanced gastric cancer (SPIRITS trial): a phase III trial. Lancet Oncol.

[12] Song Z, Wu Y, Yang J, Yang D, Fang X (2017). Progress in the treatment of advanced gastric cancer. Tumour Biol.

[13] Lauren P (1965). The two histological main types of gastric carcinoma: diffuse and so-called intestinal-type carcinoma. An attempt at a histo-clinical classification. Acta Pathol Microbiol Scand.

[14] Flejou JF (2011). WHO classification of digestive tumors: the fourth edition. Ann Pathol.

[15] Cancer Genome Atlas Research N (2014). Comprehensive molecular characterization of gastric adenocarcinoma. Nature.

[16] Cristescu R, Lee J, Nebozhyn M, Kim KM, Ting JC, Wong SS, Liu J, Yue YG, Wang J, Yu K (2015). Molecular analysis of gastric cancer identifies subtypes associated with distinct clinical outcomes. Nat Med.

[17] Liu J, Tian Z, Liu T, Wen D, Ma Z, Liu Y, Zhu J (2021). CHSY1 is upregulated and acts as tumor promotor in gastric cancer through regulating cell proliferation, apoptosis, and migration. Cell Cycle.

[18] Izumikawa T, Uyama T, Okuura Y, Sugahara K, Kitagawa H (2007). Involvement of chondroitin sulfate synthase-3 (chondroitin synthase-2) in chondroitin polymerization through its interaction with chondroitin synthase-1 or chondroitin-polymerizing factor. Biochem J.

[19] Yada T, Sato T, Kaseyama H, Gotoh M, Iwasaki H, Kikuchi N, Kwon YD, Togayachi A, Kudo T, Watanabe H (2003). Chondroitin sulfate synthase-3. Molecular cloning and characterization. J Biol Chem.

[20] Wu ZY, He YQ, Wang TM, Yang DW, Li DH, Deng CM, Cao LJ, Zhang JB, Xue WQ, Jia WH (2021). Glycogenes in oncofetal chondroitin sulfate biosynthesis are differently expressed and correlated with immune response in placenta and colorectal cancer. Front Cell Dev Biol.

[21] Yu G, Wang LG, Han Y, He QY (2012). clusterProfiler: an R package for comparing biological themes among gene clusters. OMICS.

[22] Fairfax BP, Taylor CA, Watson RA, Nassiri I, Danielli S, Fang H, Mahe EA, Cooper R, Woodcock V, Traill Z (2020). Peripheral CD8(+) T cell characteristics associated with durable responses to immune checkpoint blockade in patients with metastatic melanoma. Nat Med.

[23] Moreira AM, Pereira J, Melo S, Fernandes MS, Carneiro P, Seruca R, Figueiredo J (2020). The extracellular matrix: an accomplice in gastric cancer development and progression. Cells.

[24] Yan G, An Y, Xu B, Wang N, Sun X, Sun M (2021). Potential impact of ALKBH5 and YTHDF1 on tumor immunity in colon adenocarcinoma. Front Oncol.

[25] Chen Y, Li ZY, Zhou GQ, Sun Y (2021). An immune-related gene prognostic index for head and neck squamous cell carcinoma. Clin Cancer Res.

[26] Yang Z, Yan G, Zheng L, Gu W, Liu F, Chen W, Cui X, Wang Y, Yang Y, Chen X (2021). YKT6, as a potential predictor of prognosis and immunotherapy response for oral squamous cell carcinoma, is related to cell invasion, metastasis, and CD8+ T cell infiltration. Oncoimmunology.

[27] Guo JN, Chen D, Deng SH, Huang JR, Song JX, Li XY, Cui BB, Liu YL (2022). Identification and quantification of immune infiltration landscape on therapy and prognosis in left- and right-sided colon cancer. Cancer Immunol Immunother.

[28] Lu H, Feng Y, Hu Y, Guo Y, Liu Y, Mao Q, Xue W (2020). Spondin 2 promotes the proliferation, migration and invasion of gastric cancer cells. J Cell Mol Med.

[29] Liu JZ, Hu YL, Feng Y, Jiang Y, Guo YB, Liu YF, Chen X, Yang JL, Chen YY, Mao QS (2020). BDH2 triggers ROS-induced cell death and autophagy by promoting Nrf2 ubiquitination in gastric cancer. J Exp Clin Cancer Res.

[30] Zhu B, Chen JJ, Feng Y, Yang JL, Huang H, Chung WY, Hu YL, Xue WJ (2021). DNMT1-induced miR-378a-3p silencing promotes angiogenesis via the NF-kappaB signaling pathway by targeting TRAF1 in hepatocellular carcinoma. J Exp Clin Cancer Res.

[31] Tang Y, Zhou C, Li Q, Cheng X, Huang T, Li F, He L, Zhang B, Tu S (2022). Targeting depletion of myeloid-derived suppressor cells potentiates PD-L1 blockade efficacy in gastric and colon cancers. Oncoimmunology.

[32] Geeleher P, Cox N, Huang RS (2014). pRRophetic: an R package for prediction of clinical chemotherapeutic response from tumor gene expression levels. PLoS ONE.

[33] Tan Z (2019). Recent advances in the surgical treatment of advanced gastric cancer: a review. Med Sci Monit.

[34] Bonelli P, Borrelli A, Tuccillo FM, Silvestro L, Palaia R, Buonaguro FM (2019). Precision medicine in gastric cancer. World J Gastrointest Oncol.

[35] Anders S, Pyl PT, Huber W (2015). HTSeq—a Python framework to work with high-throughput sequencing data. Bioinformatics.

[36] Burnet M (1957). Cancer; a biological approach. I. The processes of control. Br Med J.

[37] Dunn GP, Old LJ, Schreiber RD (2004). The three Es of cancer immunoediting. Annu Rev Immunol.

[38] Mittal D, Gubin MM, Schreiber RD, Smyth MJ (2014). New insights into cancer immunoediting and its three component phases—elimination, equilibrium and escape. Curr Opin Immunol.

[39] Li Y, Wang C, Xu M, Kong C, Qu A, Zhang M, Zheng Z, Zhang G (2017). Preoperative NLR for predicting survival rate after radical resection combined with adjuvant immunotherapy with CIK and postoperative chemotherapy in gastric cancer. J Cancer Res Clin Oncol.

[40] Zhao S, Mi Y, Guan B, Zheng B, Wei P, Gu Y, Zhang Z, Cai S, Xu Y, Li X (2020). Tumor-derived exosomal miR-934 induces macrophage M2 polarization to promote liver metastasis of colorectal cancer. J Hematol Oncol.

[41] Dan H, Liu S, Liu J, Liu D, Yin F, Wei Z, Wang J, Zhou Y, Jiang L, Ji N (2020). RACK1 promotes cancer progression by increasing the M2/M1 macrophage ratio via the NF-kappaB pathway in oral squamous cell carcinoma. Mol Oncol.

[42] Li W, Zhang X, Wu F, Zhou Y, Bao Z, Li H, Zheng P, Zhao S (2019). Gastric cancer-derived mesenchymal stromal cells trigger M2 macrophage polarization that promotes metastasis and EMT in gastric cancer. Cell Death Dis.

[43] Guo X, Liang X, Wang Y, Cheng A, Zhang H, Qin C, Wang Z (2021). Significance of tumor mutation burden combined with immune infiltrates in the progression and prognosis of advanced gastric cancer. Front Genet.

[44] Wei XL, Xu JY, Wang DS, Chen DL, Ren C, Li JN, Wang F, Wang FH, Xu RH (2021). Baseline lesion number as an efficacy predictive and independent prognostic factor and its joint utility with TMB for PD-1 inhibitor treatment in advanced gastric cancer. Ther Adv Med Oncol.

[45] Pietrantonio F, Randon G, Di Bartolomeo M, Luciani A, Chao J, Smyth EC, Petrelli F (2021). Predictive role of microsatellite instability for PD-1 blockade in patients with advanced gastric cancer: a meta-analysis of randomized clinical trials. ESMO Open.

[46] Park SY, Piao Y, Jeong KJ, Dong J, de Groot JF (2016). Periostin (POSTN) regulates tumor resistance to antiangiogenic therapy in glioma models. Mol Cancer Ther.

[47] Sun W, Qin Y, Wang Z, Dong W, He L, Zhang T, Zhang H (2021). The NEAT1_2/miR-491 axis modulates papillary thyroid cancer invasion and metastasis through TGM2/NFkappab/FN1 signaling. Front Oncol.

[48] Li Y, Sun R, Zhao X, Sun B (2021). RUNX2 promotes malignant progression in gastric cancer by regulating COL1A1. Cancer Biomark.

[49] Ding YL, Sun SF, Zhao GL (2021). COL5A2 as a potential clinical biomarker for gastric cancer and renal metastasis. Medicine.

[50] Schoenfeld AJ, Hellmann MD (2020). Acquired resistance to immune checkpoint inhibitors. Cancer Cell.

[51] Bagchi S, Yuan R, Engleman EG (2021). Immune checkpoint inhibitors for the treatment of cancer: clinical impact and mechanisms of response and resistance. Annu Rev Pathol.

[52] Murciano-Goroff YR, Warner AB, Wolchok JD (2020). The future of cancer immunotherapy: microenvironment-targeting combinations. Cell Res.

