# Supplementary figures and images for: CHSY3 promotes proliferation and migration in gastric cancer and is associated with immune infiltration
Source: J Transl Med. 2023 Jul 17;21:474. doi: 10.1186/s12967-023-04333-x (PMC10351153; doi:10.1186/s12967-023-04333-x)

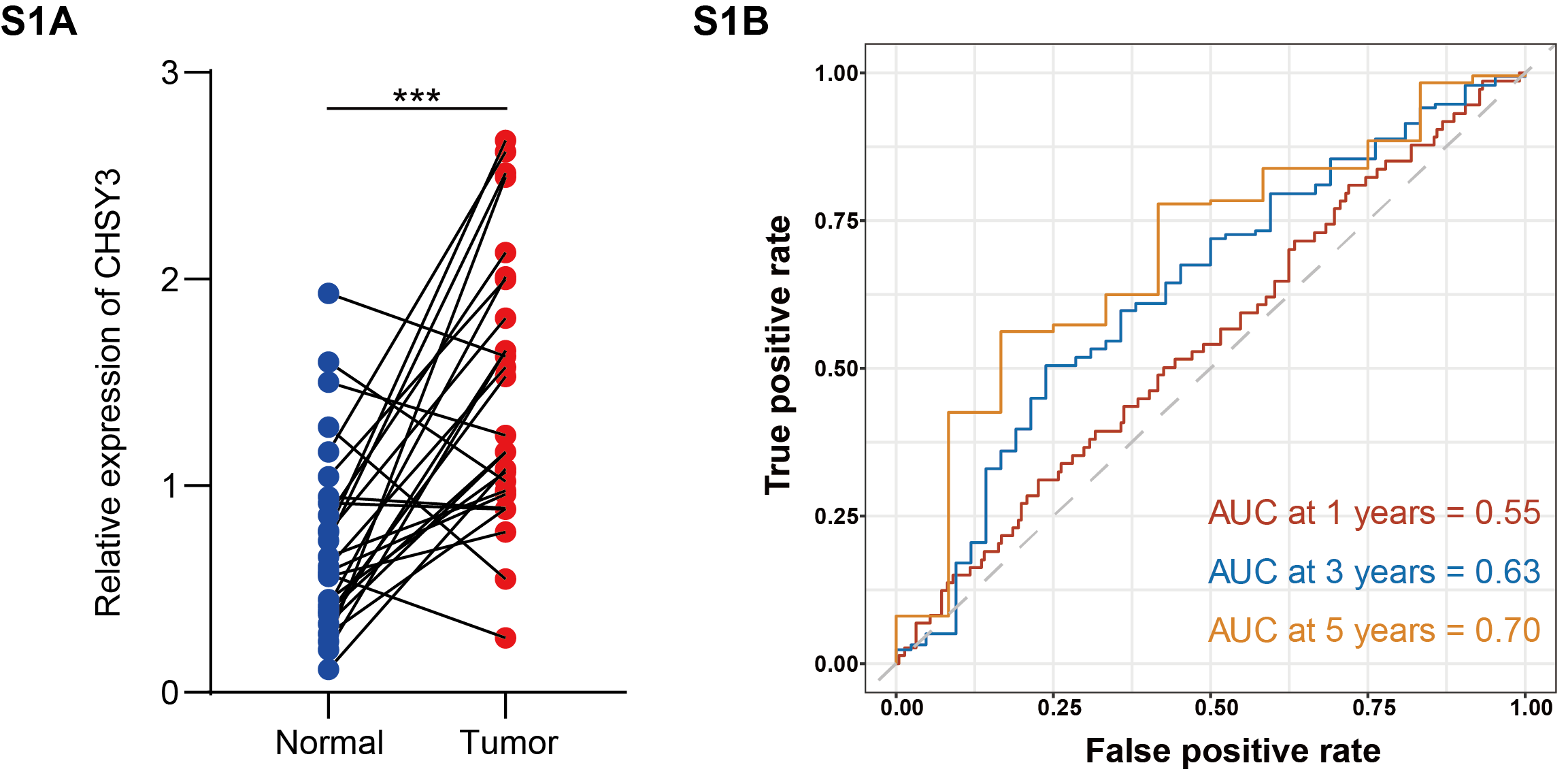

Supplement: Supplementary file 1 — Additional file 1: Figure S1. Expression of CHSY3 and ROC curve. Relative expression levels of CHSY3 in 27 pairs of gastric cancer tissues and matched paracancerous normal tissues in the TCGA database (A). The 1-year, 3-year, and 5-year ROC curves of CHSY3 (B). *P < 0.05; **P < 0.01; ***P < 0.001. [file 12967_2023_4333_MOESM1_ESM.tif]

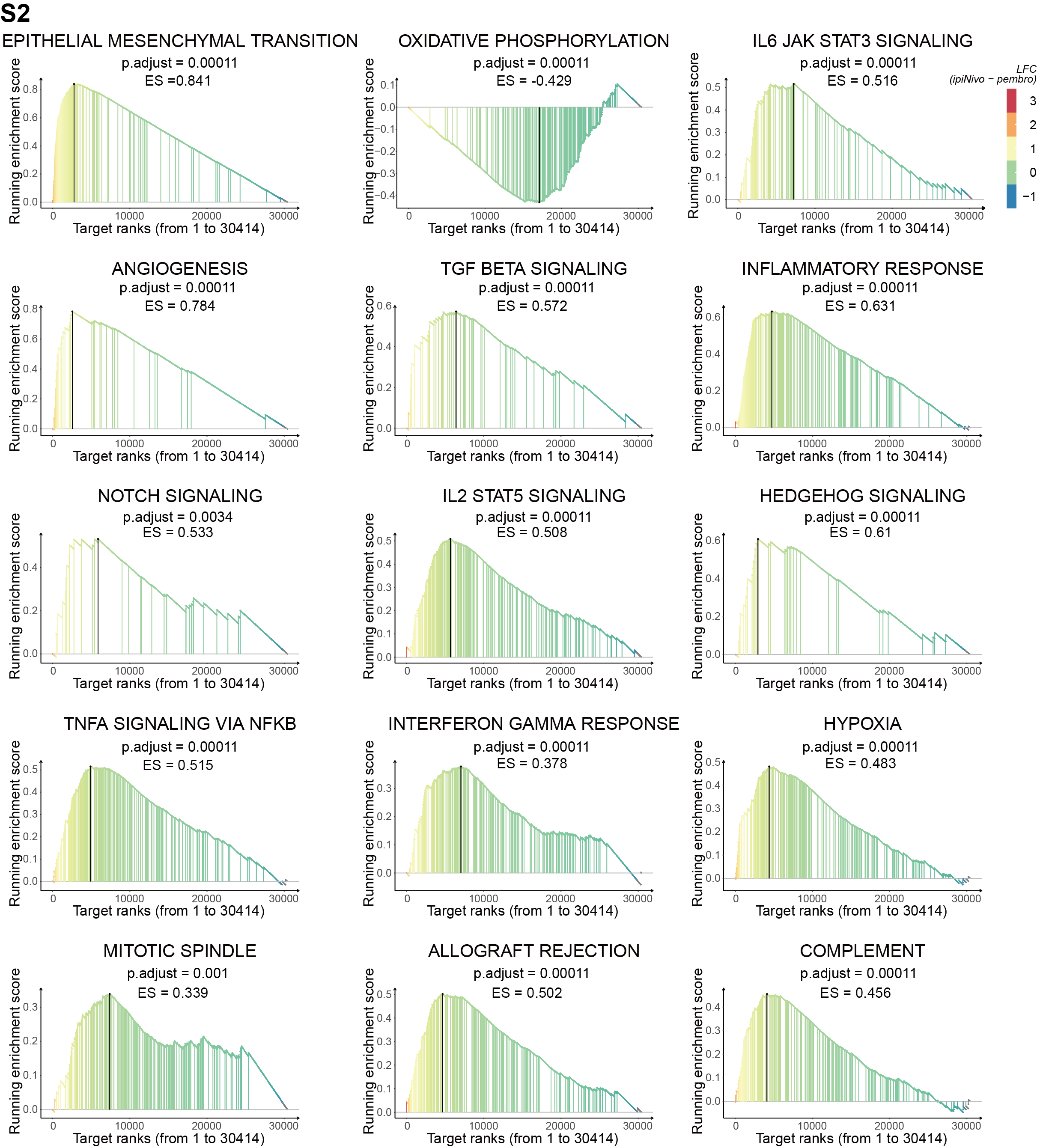

Supplement: Supplementary file 2 — Additional file 2: Figure S2. GSEA analysis of CHSY3. [file 12967_2023_4333_MOESM2_ESM.tif]

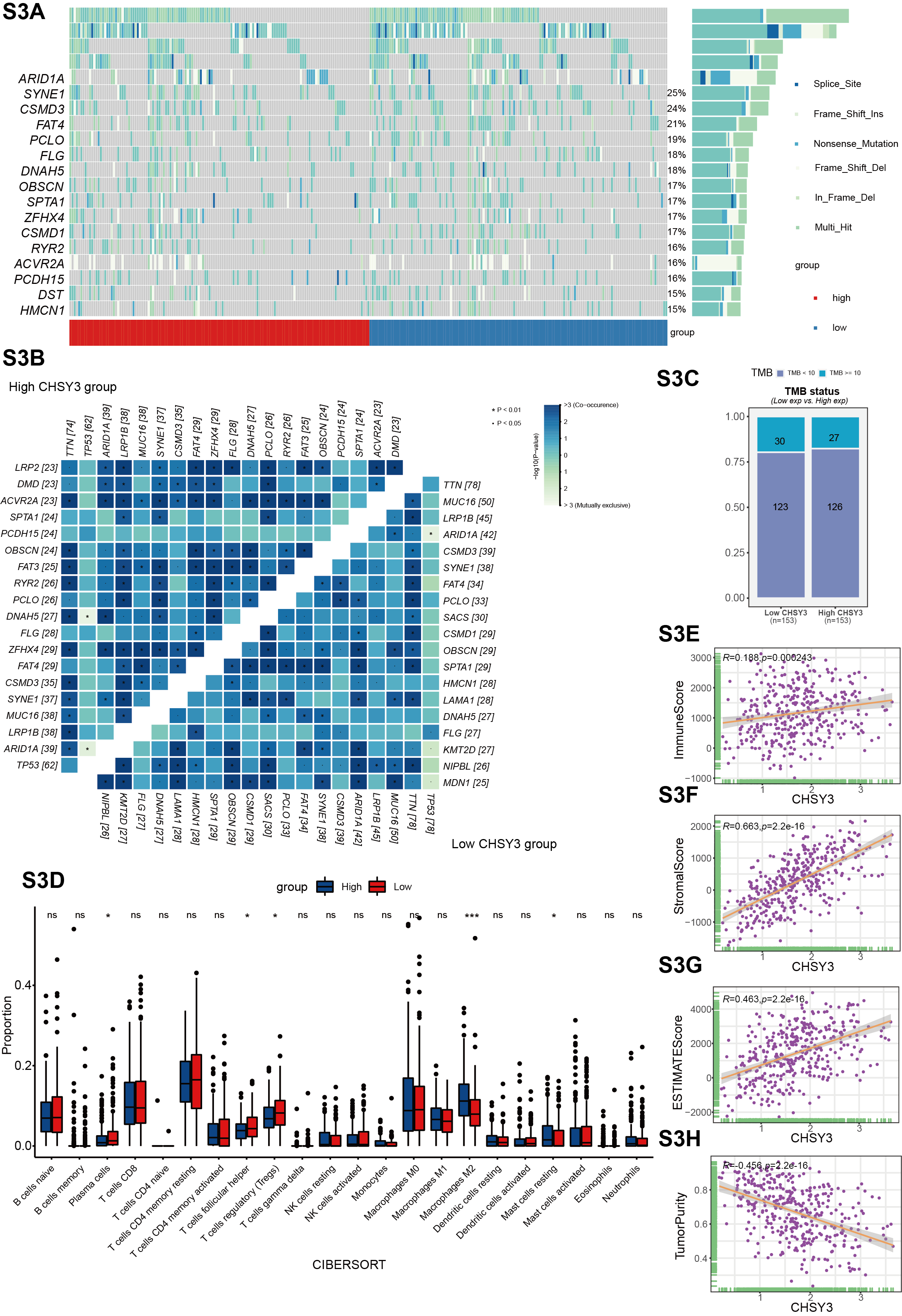

Supplement: Supplementary file 3 — Additional file 3: Figure S3. Mutation characteristics and immunological characteristics of CHSY3. Waterfall diagram showing CHSY3 mutation characteristics (A). Correlation between the top 20 genes with mutation frequency in different CHSY3 expression subgroups (B). Association between different CHSY3 expression subgroups and TMB with a threshold of 10 muts/Mb (C). Cibersort analysis of different immune cell proportions (D). Correlation of CHSY3 expression with immune score (E), stromal score (F), ESTIMATE score (G), and tumor purity (H). *P < 0.05; **P < 0.01; ***P < 0.001. [file 12967_2023_4333_MOESM3_ESM.tif]

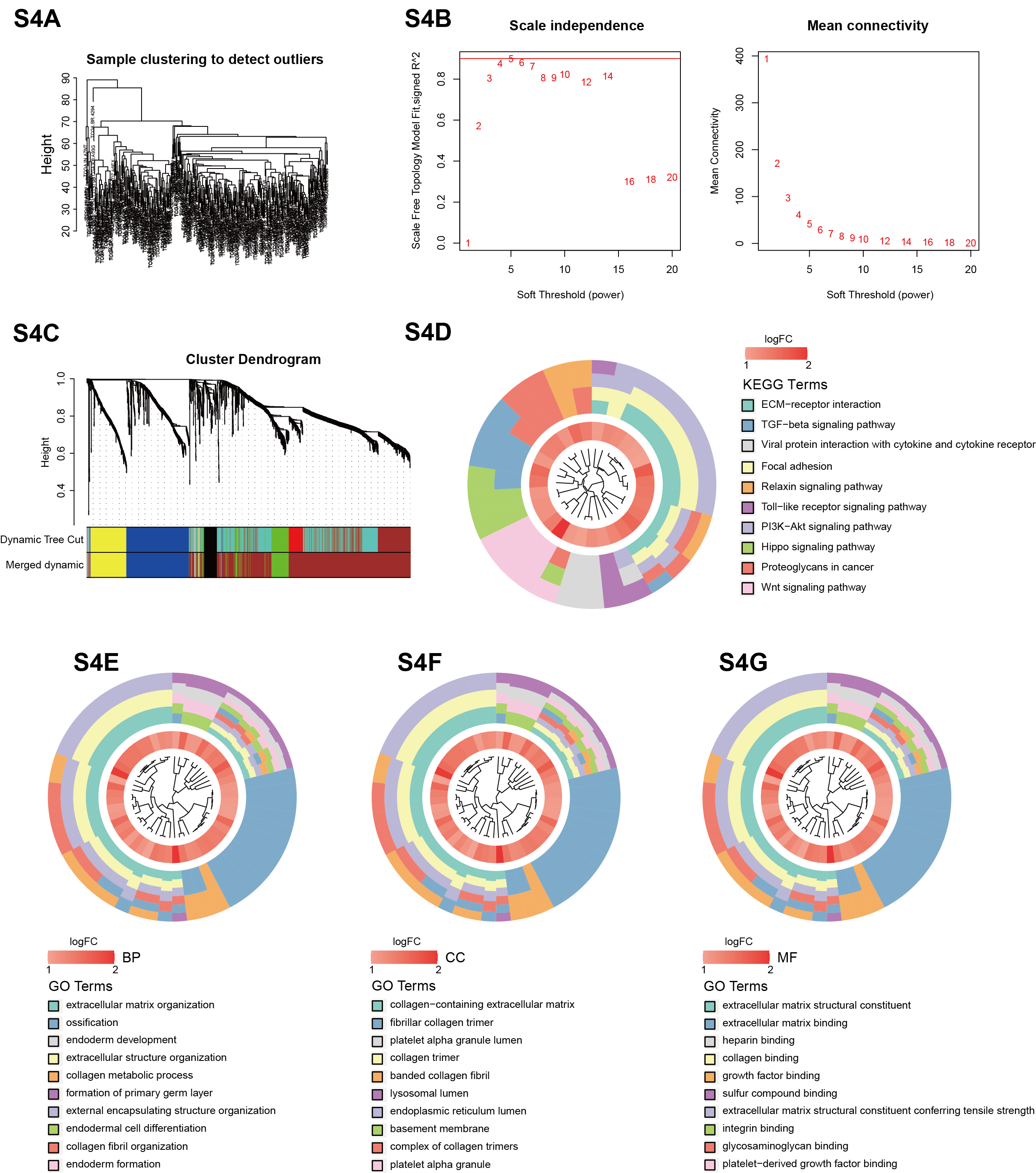

Supplement: Supplementary file 4 — Additional file 4: Figure S4. WGCNA and functional analysis. GC sample clustering in the TCGA database (A). Topological network analysis to identify optimal soft thresholds (B). Module identification (C). Kyoto encyclopedia of genes and genomes analysis (D). Gene ontology analysis (E–G). [file 12967_2023_4333_MOESM4_ESM.tif]

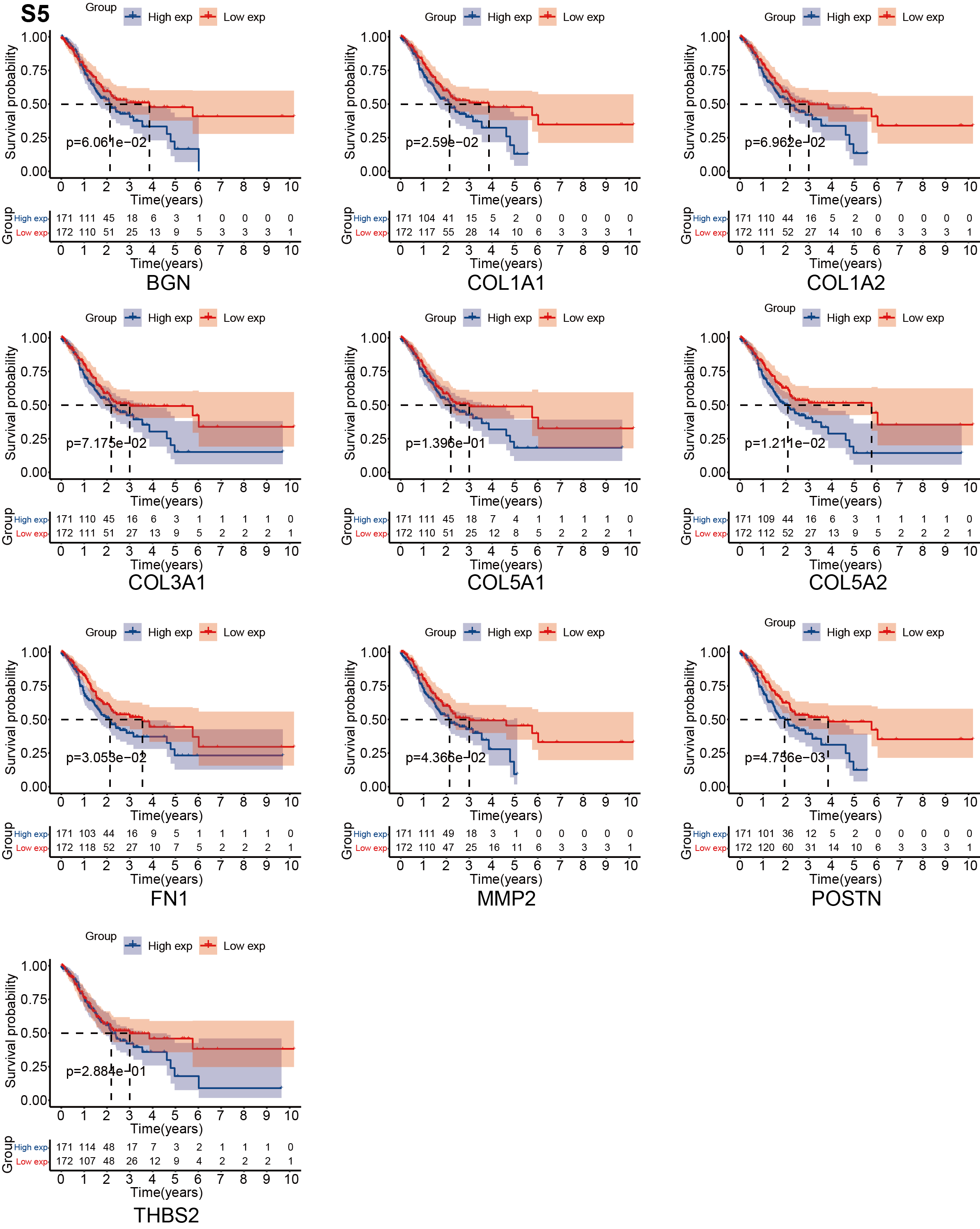

Supplement: Supplementary file 5 — Additional file 5: Figure S5. Kaplan–Meier survival curve analysis of 10 Hub genes. [file 12967_2023_4333_MOESM5_ESM.tif]

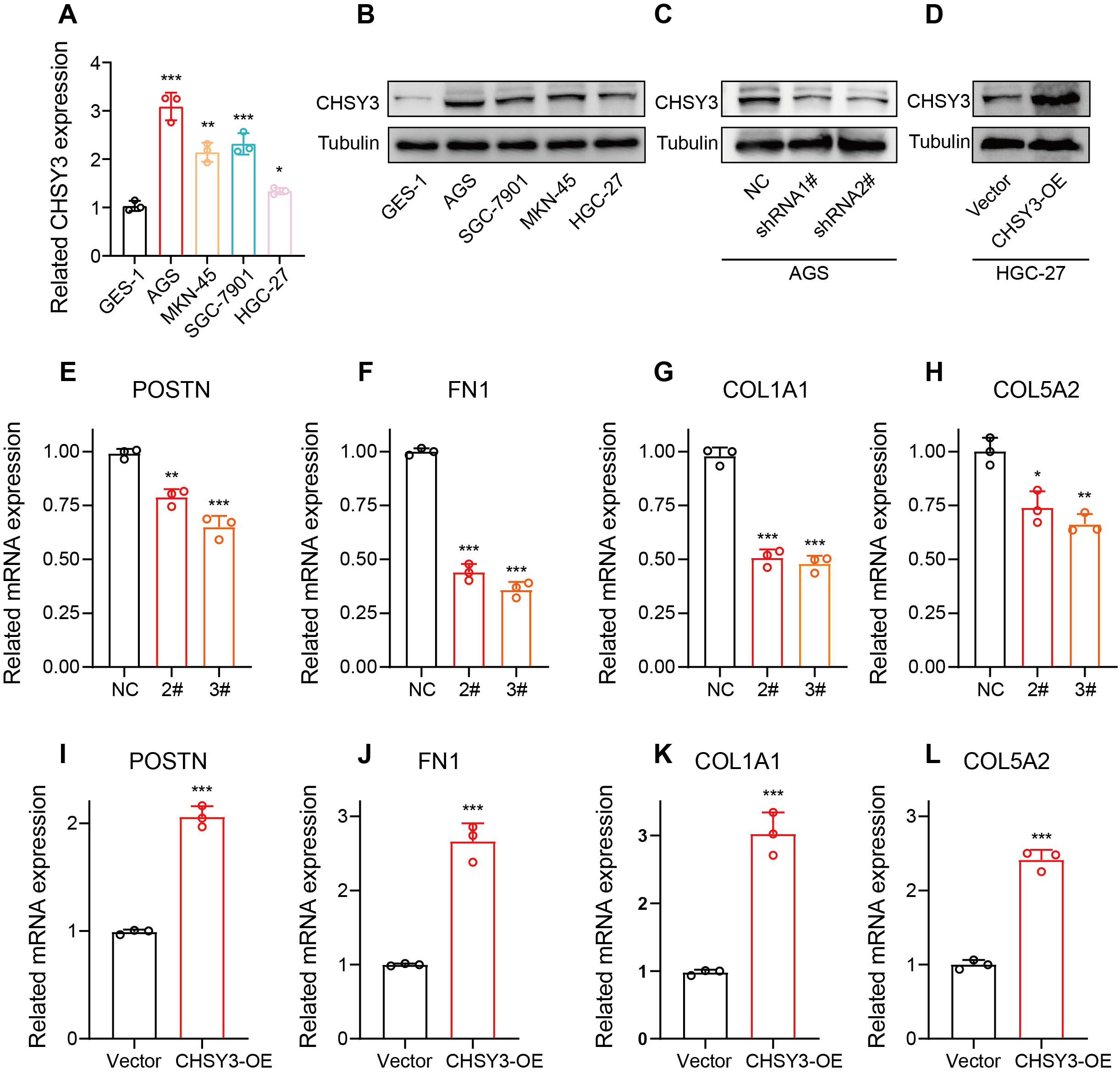

Supplement: Supplementary file 6 — Additional file 6: Figure S6. Verification of CHSY3 expression. The expression of CHSY3 in gastric cancer cell lines was analyzed by qRT-PCR and Western blot (A, B). Western blot to verify CHSY3 knockdown efficiency and overexpression efficiency (C, D). Relative mRNA expression of POSTN, FN1, COL1A1 and COL5A2 in AGS cells after CHSY3 knockdown (E–H). Relative mRNA expression of POSTN, FN1, COL1A1 and COL5A2 after overexpression of CHSY3 in HGC-27 cells (I–L). *P < 0.05; **P < 0.01; ***P < 0.001. [file 12967_2023_4333_MOESM6_ESM.tif]

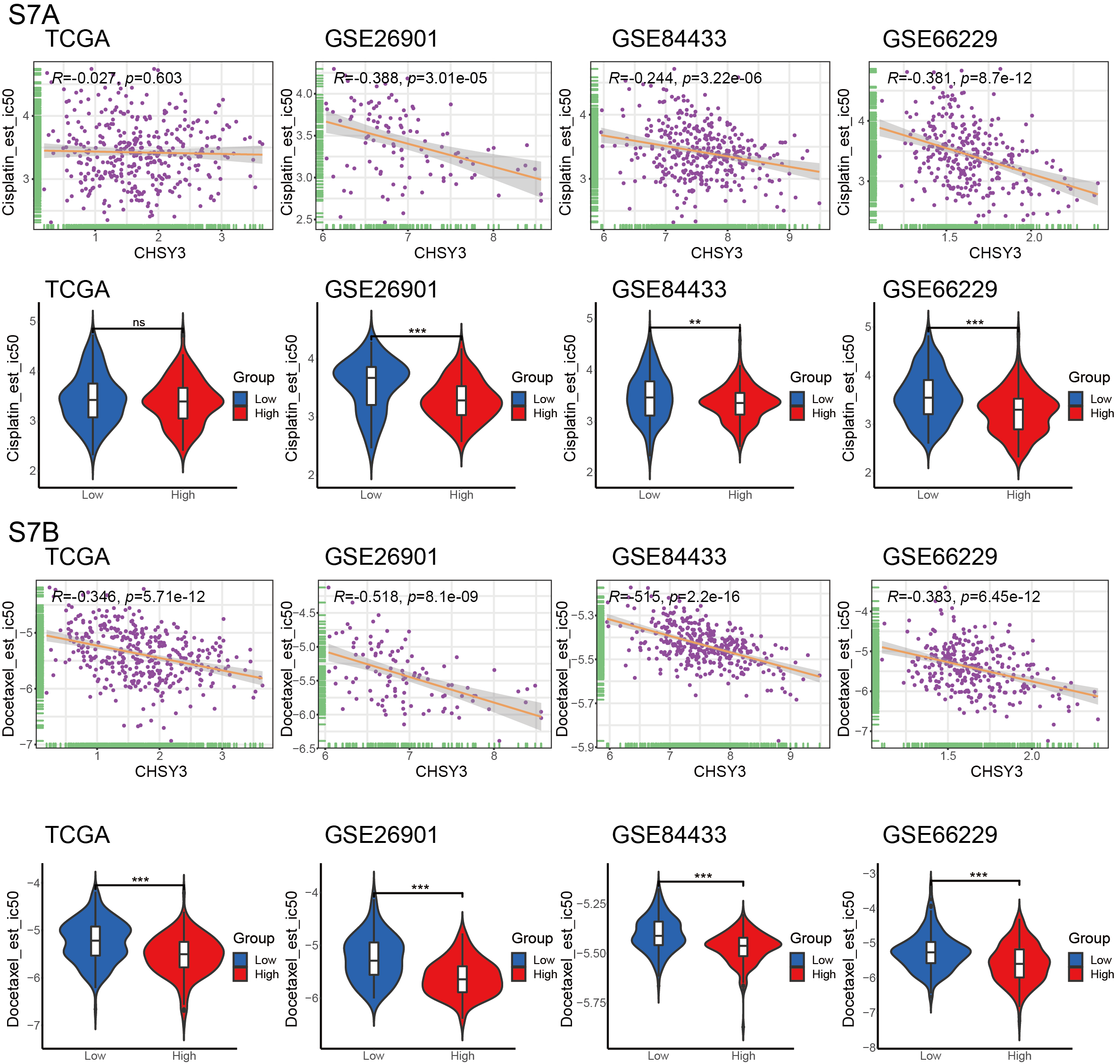

Supplement: Supplementary file 7 — Additional file 7: Figure S7. Analysis of CHSY3 expression and chemotherapeutic drug sensitivity. The R package ‘pRRophetic’ analyzed the relationship between CHSY3 expression and cisplatin sensitivity (A). Relationship between CHSY3 and docetaxel sensitivity (B). *P < 0.05; **P < 0.01; ***P < 0.001. [file 12967_2023_4333_MOESM7_ESM.tif]
